# Moving beyond button presses to enhance the reliability of congruency tasks

**DOI:** 10.3758/s13428-025-02740-0

**Published:** 2025-07-02

**Authors:** Christopher D. Erb, Samara Morrison, Alexandra Nicholson-Brown

**Affiliations:** 1 School of Psychology, University of Auckland, 23 Symonds Street, Building 302, Auckland, 1010 New Zealand; 2 HIT Lab NZ, University of Canterbury, 69 Creyke Road, John Britten Building, Christchurch, 8041 New Zealand

**Keywords:** Congruency tasks, Developmental differences, Hand tracking, Individual differences, Split-half reliability

## Abstract

Developmental and individual differences in conflict processing are commonly assessed with button-press measures of response time and accuracy in congruency tasks such as the Eriksen flanker task. However, when the reliability of response time and accuracy congruency effects are reported, the measures frequently fall short of the reliability levels needed to draw firm conclusions. In Study 1, we re-analyzed data from nine hand-tracking experiments. We found that reaching behavior generated high congruency effect reliabilities across different age groups (children, young adults, and older adults) in arrow versions of the flanker task, with split-half reliabilities often above .85 in response time, initiation time, movement time, or movement curvature. Adult performance of the Stroop task revealed congruency effect reliabilities ranging from .76 in movement curvature to .89 in initiation time. In contrast, the Simon task revealed a good reliability estimate in movement curvature for children (.84) and acceptable estimates in response time (.72), movement time (.76), and curvature (.75) for adults. Study 2 tested the reliability of a release-and-press version of the arrow flanker task using a keyboard in one condition and a touchscreen in another condition (*N* = 51). The keyboard condition generated high congruency effect reliability estimates in response time (.91), initiation time (.94), and movement time (.88), while the touchscreen condition generated high-reliability estimates in response time (.85) and initiation time (.94), and a lower estimate for movement time (.78). These results indicate that the reliability of congruency tasks can be enhanced by moving beyond traditional button-press measures of performance.

**Supplementary Information:**

The online version contains supplementary material available at 10.3758/s13428-025-02740-0.

## Introduction

Cognitive psychology has long relied on button-press measures of response time (RT) and accuracy in congruency tasks to assess developmental and individual differences in conflict processing. These tasks include the Eriksen flanker task (Eriksen & Eriksen, 1974), the Simon task (Simon, 1969), and the Stroop task (Stroop, 1935). For example, in the version of the flanker task developed by Stoffels and Van der Molen (1988) and featured in the NIH Toolbox: Cognition Battery (Zelazo et al., 2014), participants are presented with an array of arrows and are instructed to respond according to the direction cued by the centermost arrow by pressing a left or right response button. On congruent trials, the centermost arrow cues the same response as the surrounding distractor arrows (< < < < <), while the centermost arrow cues a different response than the surrounding arrows on incongruent trials (< < > < <). Performance on congruency tasks is traditionally assessed in terms of the size of the *congruency effect* observed in RT and accuracy, calculated by computing a difference score (e.g., average RT on incongruent trials minus average RT on congruent trials).

Despite the robust congruency effects observed in group-level experimental studies, investigations of developmental and individual differences frequently present worryingly low-reliability metrics (Draheim et al., 2019; Enkavi et al., 2019; Hedge et al., 2018). Following Nunnally (1964), it is generally suggested that tests need to generate reliability metrics above 0.80 for use in basic research. However, as discussed in detail by Draheim et al. (2019), the reliability metrics reported for congruency effects often fall well below this threshold. For example, in one study featuring 519 participants, Draheim et al. evaluated the split-half reliability of RT congruency effects from the color Stroop task and the arrow flanker task and observed values of 0.69 in each task. In a second study featuring 314 participants, the same tasks generated congruency effect reliabilities of 0.63 and 0.67, respectively. Similarly, in a study featuring 195 participants, Moretti et al. (2023) analyzed the split-half reliability of the RT congruency effects observed in a Simon task and a spatial Stroop task and observed congruency effect reliabilities of 0.55 and 0.59, respectively.

Research evaluating the reliability of congruency tasks across different age groups has also failed to observe adequate metrics (Erb et al., 2023; Taylor et al., 2022; Waszak et al., 2010). For example, in a study featuring more than 260 participants ranging from 6 to 88 years of age, Waszak et al. (2010) observed a Cronbach’s alpha of 0.44 when evaluating the reliability of RT congruency effects from a modified flanker task. Similarly, Erb et al. (2023) analyzed data from more than 13,000 individuals ranging from 10 to 79 years of age who completed an online version of the arrow flanker task and found that RT and accuracy congruency effects generated split-half reliability estimates of 0.36 and 0.71, respectively.

Notably, many developmental and individual difference studies using congruency tasks have historically failed to report reliability metrics, including studies from our research group (e.g., Erb & Marcovitch, 2018, 2019; Erb et al., 2020). The infrequent reporting of reliability metrics, coupled with worrying low metrics from the subset of studies that do report reliabilities, has sparked discussion of a potential reliability crisis (Rouder & Mehrvarz, 2023). As discussed in detail by Draheim et al. (2019), many of the tasks used to assess developmental and individual differences were borrowed from experimental psychology and, consequently, were designed to minimize between-participant variability and control for baseline performance differences. However, this approach contrasts with the goals of developmental and individual differences research, which seeks to understand the nature of between-participant variability. Further, RT difference scores in traditional button-press tasks fail to account for differences in speed–accuracy trade-off effects. Given (a) that individuals may differ substantially in their strategies for managing speed–accuracy trade-offs and (b) such trade-offs are likely to fluctuate over time (e.g., when participants adjust performance following an error), these differences in strategy and fluctuations in performance could eclipse differences in the target construct (Hedge et al., 2022).

To address the poor reliability of standard congruency tasks, researchers have developed new versions of the tasks that retain a focus on RT difference scores but make irrelevant information harder to ignore (Kucina et al., 2023) or that prioritize accuracy over speed (Burgoyne et al., 2023; Draheim et al., 2021). For example, Draheim et al. (2021) evaluated the reliability of adaptive congruency tasks that were designed to adjust difficulty level dynamically based on the participant’s accuracy. The difficulty level was adjusted by modulating specific aspects of the task, such as the duration of the stimulus presentation or the time allocated to respond. The researchers found that performance on accuracy-based versions of the flanker and Stroop tasks generated higher test–retest reliability measures than standard RT versions of the tasks. For example, their accuracy-based flanker task with an adaptive response deadline generated a test–retest correlation of 0.54 after outliers were excluded, whereas the RT flanker task generated a test–retest correlation of 0.23 after outliers were excluded. Similarly, the accuracy-based adaptive Stroop task generated a test–retest correlation of 0.67 after outliers were excluded, compared to a test–retest correlation of 0.46 in a standard RT Stroop task.

Building on the “double trouble” Stroop task featured in the Creyos neurocognitive battery (Creyos, “Double Trouble” n.d.), Burgoyne et al. (2023) developed “squared” versions of the flanker, Stroop, and Simon tasks in which an accuracy-based difference score is used to assess performance. These tasks also incorporate a gamified approach, featuring a points system, sound effects, and a point-and-click style interface. Notably, compared to traditional tasks, which can take up to an hour to complete, these tasks can be completed in 3 min.

The “squared” tasks also feature more conflicting information than traditional congruency tasks. For example, in the Stroop squared task, participants are shown a target stimulus with two response options below, which they can select by using their mouse to point and click on an onscreen button. Like the traditional Stroop, the stimulus (either the word “RED” or “BLUE” displayed in red or blue text) requires a response based on the color of the text rather than the meaning of the word. However, to provide a response, participants must attend to the word meaning rather than text color. For instance, if the target stimulus was “BLUE” written in red text, participants must select the response option that says “RED” regardless of the color of text the word is written in. Burgoyne et al. (2023) observed excellent split-half reliabilities in the “squared” versions of the tasks, with estimates all above 0.90.

Similarly, Kucina et al. (2023) aimed to enhance the reliability of congruency tasks using a series of modified flanker, Stroop, and Simon tasks. Rather than rejecting RT difference scores, the authors argued that the reliability of such measures could be improved by introducing task modifications that increased the magnitude of the congruency effect. Modifications included increasing the saliency of irrelevant information during trials (e.g., increased lateralization of Simon task stimuli, with task-relevant items being presented on the far sides of the screen), requiring a “double-shot” response on 1/3 of trials (in which participants were required to make a second response based on the irrelevant stimulus feature following their initial response), gamification (target stimuli are displayed as “enemies” in a “combat” style environment with boxes to hide behind), and by combining existing tasks. Results indicated that these modifications generally improved reliability. Specifically, the modified flanker task and the combined Stroop/Simon achieved high reliability (between 0.80 and 0.90) with notably fewer trials relative to traditional tasks.

Taken together, this body of work highlights the importance of developing innovative approaches to improving the reliability of congruency tasks and demonstrates how accuracy-based measures and strategic task design may enhance the efficacy of congruency tasks in differential research. In the current study, we evaluate an alternative approach to enhancing the reliability of congruency tasks. We propose that reliability may be improved by replacing traditional button-press methods with techniques that provide more insight into how the cognitive processes underlying performance unfold over time.

**Fig. 1:**
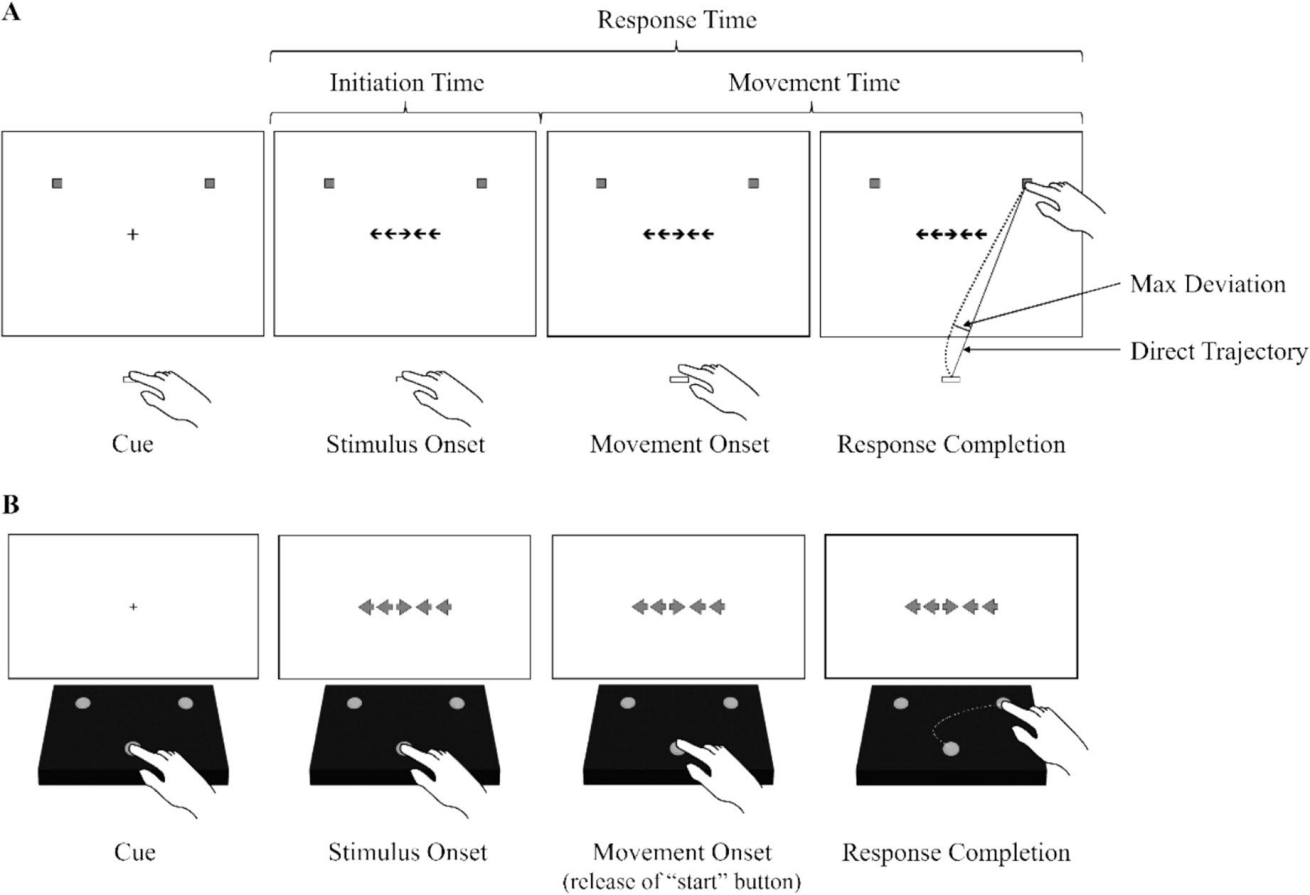
Illustration of reach-tracking and button-release-and-press versions of the flanker task. Note. **A** Illustration of a reach-tracking version of the flanker task. Participants wear a small motion-tracking sensor on their index finger and initiate each trial by resting their finger on a starting location on the table in front of them. After a cue, a stimulus array is presented, and participants respond by reaching to touch one of two response targets on the digital display. **B** Illustration of button-release-and-press method used by Smith et al. (2022). Participants initiate each trial by holding down a start button located at the bottom center of the response box. After a cue, a stimulus array is presented, and participants respond by reaching to touch one of two lateralized buttons located toward the top of the response box. This figure was adapted from Smith et al. (2022) and is presented with permission from the authors

Mouse-tracking and reach-tracking are two commonly used hand-tracking techniques. In mouse-tracking tasks, participants’ movements are tracked via a computer mouse. These tasks typically require navigating a cursor from a starting point, usually at the bottom center of the display, to designated response targets, often located in the top corners of the display (e.g., Incera & McLennan, 2016; Scherbaum & Dshemuchadse, 2020). Reach-tracking, on the other hand, involves recording participants’ three-dimensional hand movements as they reach from a starting location on a table to one of multiple response targets on a digital display (e.g., Erb et al., 2016; Erb & Marcovitch, 2018; see Fig. 1A). These hand-tracking methods afford a wide range of behavioral measures relative to traditional button-press measures, including measures of initiation time (IT; time elapsed between stimulus onset and movement onset), movement time (MT; time elapsed between movement onset and response completion), and movement curvature (CURV; a measurement of the extent to which an observed movement trajectory deviated from a direct path to the selected target, computed by dividing the length of the maximum deviation of the observed trajectory from the direct trajectory by the length of the direct trajectory).

Hand tracking presents a promising solution to the problem of poor reliability because the method allows participants to adjust their movement trajectories online, providing the opportunity to correct responses that begin on an incorrect path (sometimes referred to as partial errors; Moher & Song, 2013). Consequently, accuracy levels are often near the ceiling in hand-tracking tasks (e.g., Erb & Marcovitch, 2018, 2019), minimizing the contribution of speed–accuracy trade-off effects and maximizing the likelihood that conflict between competing responses will be captured in the temporal and spatial characteristics of reaching behavior. Thus, in contrast to approaches that seek to enhance the reliability of congruency tasks by increasing the frequency of errors (e.g., Draheim et al., 2021), hand-tracking techniques may provide an alternative route to enhanced reliability by decreasing the frequency of errors.

**Fig. 2:**
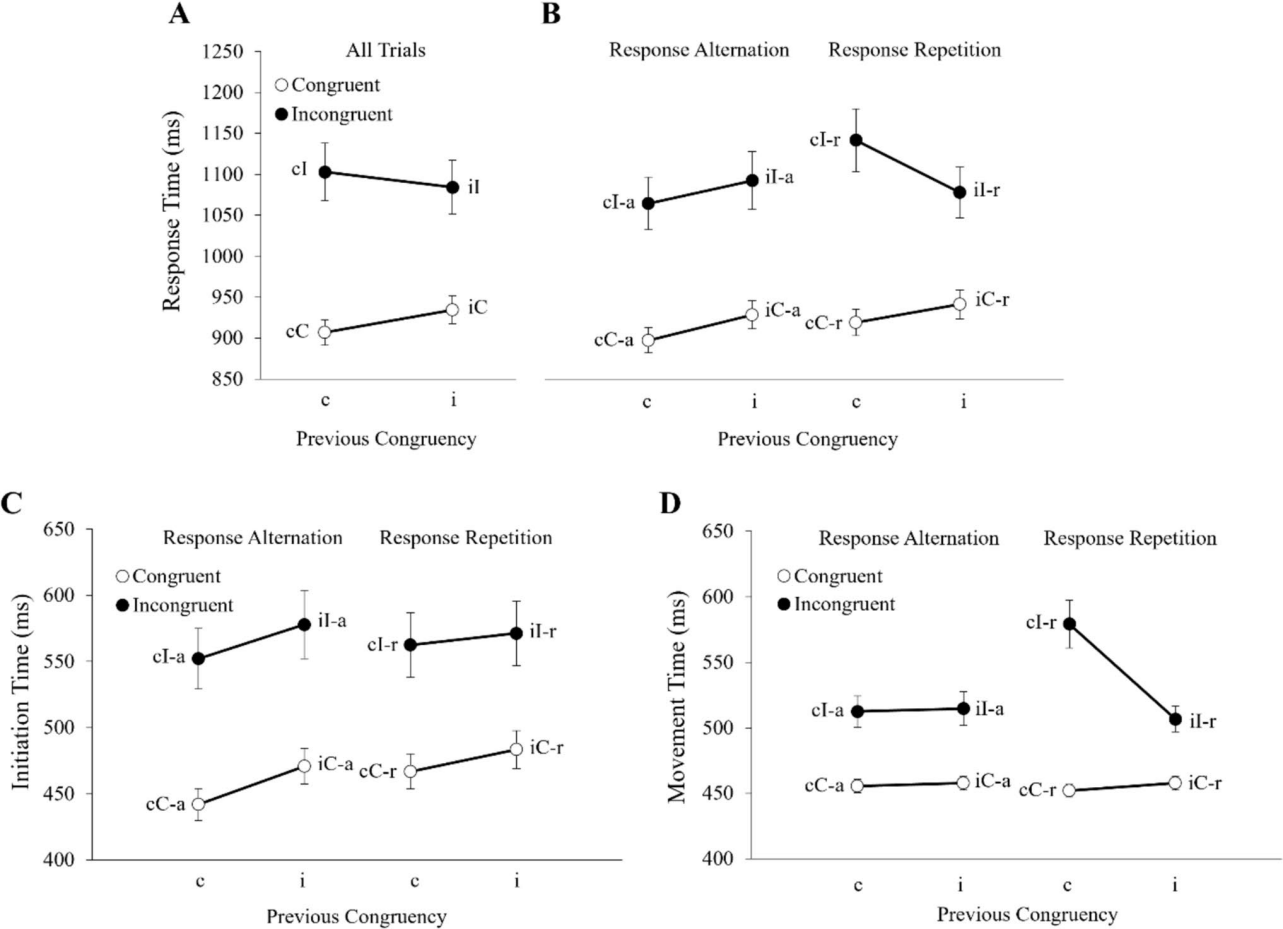
Results from reach-tracking flanker task featuring two responses. *Note.* Results from a reach-tracking version of the flanker task, which comprised 135 participants, with equal numbers of 6- to 8-year-olds, 10- to 12-year-olds, and adults (Erb & Marcovitch, 2018). The task featured arrow stimuli and two responses. **A** Response times replicated those previously reported by Gratton et al. (1992). **B** Response times also replicated those previously reported by Mayr et al. (2003) and Nieuwenhuis et al. (2006), specifically with regard to the analysis of response repetition type (alternation vs. repetition). **C** Initiation times demonstrated significant main effects of the congruency of the current trial (trial *n*) and the previous trial (trial *n*−1) congruency for both response repetition and response-alternation trials. **D** Movement times showed a significant three-way interaction between trial n congruency, trial *n*−1 congruency, and response repetition type. Notably, the pattern of effects observed in response times reflects the combination of the different patterns of effects observed in initiation times and movement times. *Error bars* show standard errors. This figure was adapted from Erb and Marcovitch (2018) and is used with the author’s consent

Hand-tracking may also help to enhance the reliability of congruency tasks by addressing an underappreciated limitation of button-press RTs; namely, the congruency effects observed in RTs appear to reflect the contribution of multiple dissociable processes that are differentially impacted by well-documented sequence effects. For example, hand-tracking investigations of the sequence effects observed in the flanker (Erb & Marcovitch, 2018; Erb et al., 2021a, b), Stroop (Erb et al., 2016), and Simon tasks (Erb & Marcovitch, 2019) demonstrate that the effects observed in RTs result from the combination of distinct patterns of effects in ITs and MTs. This point is particularly well illustrated by performance on the arrow version of the flanker task featuring two responses (see Fig. 2). Notably, ITs in the task consistently reveal main effects of current and previous congruency (see Fig. 2C), whereas MTs reveal a main effect of current congruency and a congruency sequence effect on response repetition trials but not response alternation trials (see Fig. 2D). When combined, the effects observed in ITs and MTs generate the same effect patterns previously observed in button-press versions of the task by Gratton et al., (1992; see Fig. 2A), Mayr et al. (2003), and Nieuwenhuis et al., (2006; see Fig. 2B). In light of these and similar findings from other tasks, Erb and colleagues (Erb & Marcovitch, 2018, 2019; Erb et al., 2016, 2021a, b) have argued that RTs in congruency tasks reflect the contributions of a threshold adjustment process that inhibits motor output when signals of conflict are detected and a top-down controlled selection process that biases processing in favor of task-relevant information (e.g., by focusing attention on the target arrow in the flanker task), with the notion being that ITs are more sensitive to the former, while MTs and CURVs are more sensitive to the latter (for a detailed review, see Erb et al., 2021a, b).

Thus, RT congruency effects in standard button-press tasks may also fail to generate sufficient reliability metrics because the effects reflect the contribution of multiple cognitive processes that are differentially impacted by the qualities of the previous trial. For example, RTs on congruent trials in the flanker task appear to be slowed when the preceding trial was incongruent as opposed to congruent (reflecting the pattern observed in ITs). In contrast, RTs on incongruent trials show different patterns of effects depending on whether the response of the previous trial is the same as or different than the response of the current trial (reflecting the combination of the effect patterns observed in ITs and MTs). Thus, an added benefit of assessing conflict processing with hand-tracking tasks is that it allows the reliability of specific aspects of performance to be assessed.

The notion that different hand-tracking measures can be used to identify the reliability of dissociable cognitive processes whose functioning is potentially obscured by mean RTs is consistent with results from computational modeling efforts (Miller & Ulrich, 2013). For instance, evidence-accumulation models have long proposed that mean RTs obscure the contributions of dissociable parameters underlying performance (Ratcliff & Rouder, 1998; Ratcliff et al., 2016). Researchers are increasingly exploring whether the reliability of attention tasks can be enhanced by using techniques like drift–diffusion modeling (DDM) to infer individual differences in the functioning of key model parameters (Lerche & Voss, 2017; Price et al., 2019; Rappaport et al., 2025). For example, Rappaport et al. (2025, Study 1) collected data from 381 participants who performed an arrow version of the flanker task and then used the Hierarchical Drift–Diffusion Model Toolbox (Wiecki et al., 2013) to fit a range of parameters, including drift rate and boundary separation for congruent and incongruent trials. These parameters are of particular relevance to the concerns raised regarding task reliability, given that drift rate and boundary separation are proposed to reflect evidence accumulation efficiency and speed–accuracy trade-off strategies, respectively.

For the drift rate parameters, Rappaport et al. (2025, Study 1) reported Spearman–Brown corrected split-half reliabilities of 0.84 on congruent trials and 0.72 on incongruent trials, while the boundary separation parameters generated reliabilities of 0.70 on congruent trials and 0.85 on incongruent trials. Importantly, Rappaport et al. found that these DDM parameters “related to neural and neuropsychological measures over-and-above” traditional behavioral measures like raw accuracy and the size of the RT congruency effect. Consequently, the existing literature offers at least preliminary support for the potential value of testing the reliability of different factors whose functioning is partially obscured by mean RTs.

Here, we present two studies examining the extent to which reaching tasks can overcome the methodological challenges that result in poor reliability metrics commonly found in button-press congruency tasks. Study 1 assesses the split-half reliabilities of the RT, IT, MT, and CURV congruency effects observed in 14 datasets from nine previously published experiments, reflecting a range of different tasks (flanker, Stroop, and Simon tasks) and age groups (children, young adults, and older adults). Study 2 then examines whether a simple release-and-press response method using a standard keyboard or touchscreen display can generate acceptable reliability estimates in an arrow version of the flanker task. Following Rappaport et al. (2025), we will adopt the following cutoffs from Henson (2001) when labeling split-half reliabilities at different levels: < 0.70 = poor, 0.70-0.79 = acceptable, 0.80–0.89 = good, and > 0.90 = excellent.

## Study 1

### Participants and method

Study 1 analyzed data from nine previously published experiments encompassing a diverse sample of 661 participants ranging between 5 to 75 years of age. Table 1 presents a brief description of the tasks and samples included in each of the experiments. Further details regarding the methods and participants for each experiment are available in the referenced publications. Eight of the experiments used an electromagnetic position and orientation recording system (Polhemus) to measure reaching behavior in modified versions of the flanker task (six experiments), the Stroop task (one experiment), or the Simon task (one experiment). This method involved participants reaching toward onscreen response locations with a motion-tracking sensor attached to their index finger. Each movement was initiated from a designated starting location on the table in front of the participant, and the tasks were displayed on an acrylic screen using a rear-mounted projector.

**Table 1:** Summary of datasets included in re-analysis

Experiment	Task	Responses and stimuli	Number of trials	Age group	*N*
Erb et al., (2016, Exp. 2)	Flanker	Three responses (bottom left, bottom right, top center); Three letters (A, B, K)	288	Young adults (*M* = 19.5 years, *SD* = 1.2)	40
Erb et al., (2018, Exp. 1)	Flanker	Two responses (left, right); Cartoon fish stimuli facing left or right	108	5- to 10-year-olds (*M* = 7.6 years, *SD* = 1.6)	60
Erb et al., (2018, Exp. 2)	Flanker	Two responses (left, right); Arrows facing left or right	240	Young adults (*M* = 20.1 years, *SD* = 1.3)	24
Erb and Marcovitch (2018)	Flanker	Two responses (left, right); Arrows facing left or right	144	6- to 8-year-olds (*M* = 6.9 years, *SD* = 0.8)	45
				10- to 12-year-olds (*M* = 11.0 years, *SD* = 0.8)	45
				Young adults (*M* = 18.7 years, *SD* = 1.4)	45
Erb et al. (2020)	Flanker	Two responses (left, right); Arrows facing left or right	192	Older adults (*M* = 69.0 years, *SD* = 2.8)	45
Erb et al., (2021a, b)	Flanker	Two responses (left, right); Arrows facing left or right	288	Young adults (*M* = 19.3 years, *SD* = 2.1)	135
Smith et al. (2022)	Flanker	Release-and-press button box; Two responses (left, right); Arrows facing left or right	192	6- to 8-year-olds (*M* = 7.4 years, *SD* = 0.9)	45
				Young adults (*M* = 20.7 years, *SD* = 2.0)	45
Erb et al., (2016, Exp. 1)	Stroop	Three responses (bottom left, bottom right, top center); Three words and colors (RED, GREEN, BLUE)	480	Young adults (*M* = 19.6 years, *SD* = 1.2)	24
Erb and Marcovitch (2019)	Simon	Two responses (left, right); Heart and sun icons	160	6- to 8-year-olds (*M* = 7.0 years, *SD* = 0.8)	36
				10- to 12-year-olds (*M* = 11.0 years, *SD* = 0.9)	36
				Young adults (*M* = 18.8 years, *SD* = 1.3)	36

In contrast to the studies featuring the electromagnetic tracking system, one experiment (Smith et al., 2022) investigated flanker performance using a custom-made 3-button response box (see Fig. 1B). Participants were required to press and hold a centrally located button to begin each trial. After the stimulus array appeared, they released the central button and reached to press one of two lateralized buttons located toward the top of the response box. This method allowed for the measurement of initiation time (IT; time elapsed between stimulus presentation and release of the central button), movement time (MT; time elapsed between release of the central button and the pressing of one of the lateralized buttons), and response time (RT; the time elapsed between stimulus presentation and the pressing of one of the response buttons. However, this method did not allow for reach curvature to be assessed, as movement trajectories were not recorded.

### Results

The data, analysis scripts, and Supplementary Materials for this study are available at https://osf.io/pvd7t/. The descriptive statistics for the congruency effects observed in each of the flanker datasets are presented in Table 2, while the descriptive statistics for the congruency effects observed in the Stroop and Simon datasets are presented in Table 3. Section 1 of the Supplementary Materials provides the descriptive statistics for each of the measures across all trials (congruent and incongruent). For all datasets, the first trial of each block was excluded from the analysis. All error responses and trials following an error were also excluded from analysis of RT, IT, MT, and CURV to control for post-error performance adjustments (Danielmeier & Ullsperger, 2011). Additional inclusion criteria for trials followed the criteria published in the original studies with two exceptions: the flanker and Stroop datasets from Erb et al. (2016). The primary analyses in Erb et al. (2016) excluded trials that featured a repetition of the stimulus or response features of the preceding trial to address specific hypotheses of interest, whereas the current study includes these trials, given our focus on reliability.

**Table 2:** Descriptive statistics for congruency effects observed in response time, initiation time, movement time, and curvature for flanker task datasets

Experiment	Age group	Measure	*M*	*SD*	Min	Max	Skew	Kurtosis
Erb et al., (2016, Exp. 2)	Young adults	RT	32	14	5	66	0.42	– 0.24
		IT	16	13	– 15	46	– 0.02	– 0.08
		MT	17	12	– 12	48	0.41	– 0.03
		CURV	0.040	0.033	– 0.033	0.110	0.17	– 0.21
Erb et al., (2018, Exp. 1)	5- to 10-year-olds	RT	127	235	– 65	1507	4.46	21.41
		IT	47	107	– 280	585	2.04	10.42
		MT	80	166	– 39	1217	5.63	35.10
		CURV	0.058	0.040	– 0.004	0.149	0.50	– 0.69
Erb et al., (2018, Exp. 2)	Young adults	RT	44	18	2	89	– 0.06	0.56
		IT	35	21	– 4	89	0.49	0.15
		MT	10	11	– 5	31	0.45	– 0.89
		CURV	0.041	0.033	– 0.004	0.100	0.53	– 1.08
Erb and Marcovitch (2018)	6- to 8-year-olds	RT	385	322	80	1520	1.67	2.52
		IT	217	226	– 33	1174	2.23	5.69
		MT	168	150	8	685	1.59	2.48
		CURV	0.095	0.063	– 0.003	0.294	1.23	1.55
	10- to 12-year-olds	RT	82	50	– 2	247	1.39	2.14
		IT	43	33	– 9	124	0.73	– 0.10
		MT	39	30	– 2	135	1.03	1.12
		CURV	0.07	0.036	0.004	0.179	0.38	0.25
	Young adults	RT	52	36	12	236	3.20	13.07
		IT	40	36	3	222	3.25	13.33
		MT	12	14	– 8	61	1.40	1.97
		CURV	0.043	0.032	– 0.002	0.149	1.24	1.67
Erb et al. (2020)	Older adults	RT	91	63	– 14	399	2.77	11.24
		IT	67	56	3	321	2.23	7.52
		MT	24	23	– 45	78	0.03	0.79
		CURV	0.052	0.04	0.001	0.183	1.22	1.20
Erb et al., (2021a, b)	Young adults	RT	42	23	4	209	3.42	19.61
		IT	28	23	– 8	161	1.90	7.64
		MT	14	14	– 10	77	1.16	2.33
		CURV	0.05	0.034	– 0.003	0.164	0.60	– 0.19
Smith et al. (2022)	6- to 8-year-olds	RT	322	257	89	1322	2.03	4.38
		IT	162	132	– 48	554	0.99	0.75
		MT	160	203	– 41	1083	2.78	8.82
	Young adults	RT	61	21	19	120	0.49	0.73
		IT	26	16	– 4	62	0.25	– 0.27
		MT	35	23	– 4	104	1.00	1.03

**Table 3:** Descriptive statistics for congruency effects observed in response time, initiation time, movement time and curvature for Stroop and Simon task datasets

Experiment	Task	Age group	Measure	*M*	*SD*	Min	Max	Skew	Kurtosis
Erb et al., (2016, Exp. 1)	Stroop	Young adults	RT	59	38	7	146	0.77	– 0.49
			IT	28	27	– 5	99	1.31	0.97
			MT	32	23	– 1	90	0.73	– 0.10
			CURV	0.043	0.041	– 0.005	0.132	0.70	– 0.68
Erb and Marcovitch (2019)	Simon	6- to 8-year-olds	RT	55	70	– 116	219	0.41	0.41
			IT	12	39	– 74	170	1.56	5.84
			MT	44	59	– 113	173	0.20	0.62
			CURV	0.097	0.078	– 0.025	0.278	0.60	– 0.32
		10- to 12-year-olds	RT	25	31	– 39	103	0.28	0.37
			IT	11	21	– 33	65	0.20	0.04
			MT	13	23	– 27	62	0.51	– 0.11
			CURV	0.039	0.035	– 0.036	0.112	0.37	– 0.48
		Young adults	RT	15	21	– 14	102	2.04	6.19
			IT	8	18	– 21	91	2.69	10.51
			MT	7	9	– 11	33	0.17	0.85
			CURV	0.022	0.026	– 0.020	0.091	0.88	0.38

To evaluate the potential impact of adopting a consistent filtering approach on the datasets, we conducted additional analyses in which we excluded all trials featuring RTs under 200 ms or over 2.5 s for adult participants and all trials featuring RTs under 200 ms or over 4 s for child participants. The mean and standard deviation of each participants’ overall response time on each condition were then calculated. Any trials featuring a RT greater than 3 SDs from an individual’s own mean RT for each condition were considered outliers and subsequently removed. Given that some of the datasets showed substantial deviations from a normal distribution, we also log-transformed the filtered datasets. The results of these analyses are summarized in Sect. 2 of the Supplementary Materials. We also highlight any consequential discrepancies between the original datasets and the filtered and log-transformed datasets in the results presented below.

Average error rates were generally low across all the datasets, often under 1% (e.g., Erb et al., 2020, 2021a, b). Three exceptions were noted: Erb et al., (2016, Experiment 1) observed an average error rate of 3.5% in adult participants who completed a Stroop task featuring three response options, Smith et al. (2022) observed an average error rate of 5.3% in children 6 to 8 years of age who completed a two-response version of the flanker task featuring arrow stimuli, and Erb and Marcovitch (2019) observed an average error rate of 3.6% in children 6 to 8 years of age who completed a two-response version of the Simon task.

As noted by Draheim et al. (2019), the reliability of a difference score is systematically related to the correlation between the component scores that are used to generate the difference score (e.g., RTs on congruent and incongruent trials) and the reliabilities of the component scores themselves. To examine the correlation between the component scores that were used to generate congruency effects, we correlated average performance on congruent trials with average performance on incongruent trials for each dependent measure. Table 6 in the Appendix presents these correlations for each of the flanker datasets, while Table 7 presents these correlations for the Stroop and Simon datasets.

Two major themes emerge from these correlational analyses. First, the correlations observed in the adult datasets are uniformly high for the temporal measures (RT, IT, and MT), usually above 0.90 and often above 0.95. In the child datasets, the correlations for the temporal measures are generally lower, with one dataset in the 0.60 to 0.80 range. Second, the correlations between congruent and incongruent trials are consistently lower in CURV than the temporal measures, regardless of age group or task, with most datasets showing values between 0.55 and 0.80, and two datasets showing values between 0.35 and 0.40.

Next, we calculated component reliabilities for congruent and incongruent trials separately, as well as reliabilities for the congruency effect. All data sets were analyzed using a permutation-based split-half approach, a robust method for estimating reliability (Parsons, 2021), with 5000 random splits for each study and its respective participant age groups. The analysis focused on the congruency effects of four key measures: RT, IT, MT, and CURV. The Spearman–Brown corrected reliability estimates for the component scores observed in the flanker datasets are presented in Table 8 of the Appendix, while the estimates for the Stroop and Simon datasets are presented in Table 9.

**Table 4:**
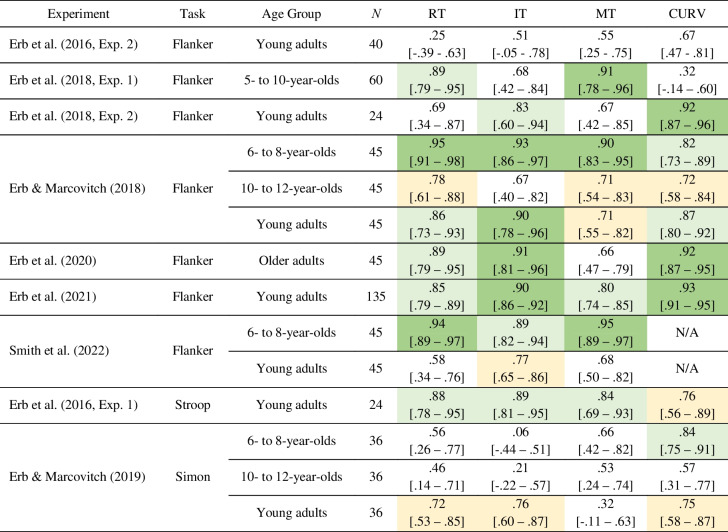
List of experiments with split-half Spearman–Brown corrected reliability estimates for response time (RT), initiation time (IT), movement time (MT), and movement curvature (CURV) congruency effects

Yellow, light green, and dark green cells indicate Spearman–Brown corrected split-half reliability estimates of 0.70 to 0.79 (acceptable), 0.80 to 0.89 (good), and 0.90 to 1.0 (excellent), respectively. 95% confidence intervals are presented in brackets.

The flanker datasets generated uniformly high-reliability estimates for each temporal measure component score, with all reliability estimates above 0.90 for RT, IT, and MT, and most estimates well above 0.95. Curvature component scores were between 0.90 and 0.97 for adult datasets, and between 0.60 and 0.88 for child and pre-adolescent datasets. The component reliability estimates in the Stroop task were uniformly high for the temporal measures (all 0.99), while curvatures generated an estimate of 0.95 for congruent trials and 0.91 for incongruent trials. The component reliability estimates in the Simon datasets were uniformly high in the temporal measures (all above 0.93), while curvatures generated estimates between 0.72 and 0.89 for congruent trials and of 0.90 or higher in incongruent trials.

Finally, we evaluated the reliability of the congruency effects observed in each measure. The Spearman–Brown corrected reliability estimates for each study are presented in Table 4. For the three datasets with high error rates, split-half reliability estimates for the error rate congruency effects observed in these datasets were also calculated, which we report below.

In general, reaching versions of the flanker task generated good to excellent reliability estimates in one or more of the reported measures. However, there were two exceptions. The three-response version of the flanker task featuring letter stimuli from Erb et al., (2016, Experiment 2) failed to generate acceptable reliability estimates for the congruency effects observed in any of the measures. Similarly, the sample of adult participants from Smith et al. (2022), who completed an arrow version of the flanker task using a release-and-press button box, only showed an acceptable reliability estimate for the congruency effect observed in IT (0.77). Of the remaining eight flanker datasets, all but one showed acceptable to excellent reliability estimates in RT congruency effects (ranging from 0.78 to 0.95), six showed acceptable to excellent reliability estimates in IT congruency effects (ranging from 0.83 to 0.93), six showed acceptable to excellent reliability estimates in MT congruency effects (ranging from 0.71 to 0.95), and six showed acceptable to excellent reliability estimates in CURV congruency effects (ranging from 0.72 to 0.93), with no CURV data available in one of the datasets (Smith et al., 2022, 6- to 8-year-olds). For the Smith et al. (2022) 6- to 8-year-old group, error rate reliability was excellent, generating a reliability estimate of 0.93.

The dataset from the Stroop task demonstrated good-to-excellent reliability across RT, IT, and MT congruency effects (0.84 or higher), and acceptable reliability in CURV (0.76). Analysis of the error rate reliability also revealed good reliability with a coefficient of 0.82. Simon task reliability was generally low across child age groups for all measures, with the notable exception of CURV congruency effects, which displayed a reliability estimate of 0.84 for the 6- to 8-year-olds. In the young adult age group, the congruency effect observed in the Simon task was in the acceptable range for RT (0.72), IT (0.76), and CURV (0.75), but not MT (0.32). Analysis of error rate reliability in the sample of 6- to 8-year-olds generated a poor reliability estimate of 0.59.

Analysis of the filtered and log-transformed datasets revealed two substantial differences from the reliability analyses presented here. First, the congruency effects observed in IT and MT in child participants from Erb et al., (2018, Exp. 1) dropped considerably in our analysis of filtered, log-transformed data, with RT at 0.62 (from the original 0.89), IT at 0.39 (from the original 0.68), and MT at 0.31 (from the original 0.91). This variability in estimates likely reflects the relatively small number of trials collected in the experiment, along with the disproportionate exclusion of incongruent trials during the filtering process. Consequently, the results of the Erb et al., (2018, Exp. 1) dataset should be interpreted with caution. The other notable difference concerned the congruency effects observed in IT and RT in adults performing the Simon task (Erb & Marcovitch, 2019). In our analysis of the filtered, log-transformed data, the IT reliability estimate dropped to 0.51 (from 0.76 in the original dataset), while the RT reliability estimate dropped from 0.72 in the original dataset to 0.67. The reliability estimate for CURV remained similar at 0.76 (0.75 in the original dataset).

### Discussion: Study 1

Our analysis of nine previously published experiments using hand-tracking methods across different tasks and age groups provides a promising approach to enhancing the reliability of congruency tasks. Our findings demonstrate that hand-tracking versions of the flanker and Stroop tasks routinely generated acceptable to excellent RT reliability metrics, suggesting that hand-tracking methods provide more reliable measures of performance than traditional button-press methods. However, two notable exceptions were observed within the flanker datasets we evaluated, potentially attributable to differences in stimuli and methodology: the letter version of the flanker task featured in Erb et al. (2016) generated a low-reliability estimate for the RT congruency effect, as did adult performance of the arrow version of flanker task featured in Smith et al. (2022) that utilized a novel release-and-press button box method. RTs in the Simon task generated an acceptable reliability estimate in adults, but not in 6- to 8-year-olds or 10- to 12-year-olds.

IT, MT, and CURV generated congruency effect reliability estimates ranging from acceptable to excellent across most of the flanker and Stroop datasets analyzed. Interestingly, the flanker and Stroop congruency effects observed in these measures were generally comparable in reliability to the congruency effects observed in RT, indicating that the reliability of RT effects was not substantially impacted by the combination of different effect patterns in ITs and MTs. Notably, adults tended to show higher reliability estimates in CURV than MT in the flanker task, whereas children’s performance did not reveal this trend. This difference may reflect increased variation in the extent to which adults alter their speed after correcting the direction of their reach.

In the Simon task, MT congruency effects generated poor reliability estimates for each of the age groups tested. The congruency effects observed in young adults generated acceptable reliability estimates in RT, IT, and CURV. However, none of the measures revealed acceptable reliability estimates in 10- to 12-year-olds, and CURV was the sole measure to generate an acceptable reliability estimate in 6- to 8-year-olds. Thus, the congruency effect observed in the Simon task appears to be less reliable than that of the arrow version of the flanker task, with MT providing a particularly unreliable measure of Simon performance.

The results of Study 1 indicate that hand-tracking methodologies may offer a promising avenue for enhancing the reliability of congruency tasks. However, Study 1 presented several limitations that merit acknowledgment. First, most of the datasets evaluated in the current study featured relatively small sample sizes for studies investigating individual differences. Consequently, it is likely that the reported reliability estimates have not stabilized (Schönbrodt & Perugini, 2013). Second, the majority of datasets analyzed in the current study were collected with electromagnetic reach-tracking systems. These systems, while providing excellent spatial and temporal resolutions, are costly and difficult to work with given their sensitivity to ferromagnetic metals. Consequently, electromagnetic systems present significant barriers to adoption. Our analyses indicate that the release-and-press button box used by Smith et al. (2022) presents an affordable, easy-to-use alternative to electromagnetic tracking that provides excellent reliability metrics in child participants and an acceptable reliability estimate for the IT congruency effect in adults (0.77).

Given that the CURV congruency effects observed in the electromagnetic tracking datasets tended to generate higher reliability estimates in adults than MT congruency effects, the release-and-press button box may be less effective for capturing reliable effects in adults than methods that capture the spatial characteristics of responses. It is important to highlight, however, that using a release-and-press approach to separating RTs into different temporal measures allowed for a more reliable measure of performance – namely, IT – to be identified in adults (Smith et al., 2022). Consequently, the release-and-press approach may still be preferable when compared to traditional button-press methods.

However, further research is needed to test whether the release-and-press approach can be modified to provide stronger reliability estimates in adults. For example, participants in the Smith et al. (2022) study were encouraged to maintain fixation on the digital display during the task because their looking behavior was recorded via a screen-mounted eye-tracker. It is therefore possible that the relatively low-reliability estimates observed in adult performance in the Smith et al. study stemmed from a reliance on peripheral vision when reaching for response targets, resulting in more variable movements. Notably, adults spent a greater percentage of each trial looking at the display (*M* = 99%) than children (*M* = 86%), suggesting that children were more likely to look down at the button box when reaching for a response target. Thus, the release-and-press method might generate more reliable estimates of congruency effects in adults when eye movements are unconstrained.

## Study 2

The results of Study 1 demonstrated that hand-tracking versions of the flanker task routinely generate acceptable to excellent congruency effect reliability estimates. Given that electromagnetic reach-tracking systems are expensive and difficult to use, it is important to determine whether low-cost alternatives can provide comparable reliability estimates. In light of the promising reliability estimates observed in a release-and-press flanker task from Smith et al. (2022), our second study investigated whether the release-and-press method can be used to enhance reliability estimates in an arrow version of the flanker task completed under two conditions: one using a standard keyboard and the other using a touchscreen display. In the keyboard condition, participants began each trial by pressing and holding a start key. After the stimulus array appeared, participants responded by releasing the start key and pressing the key to the left or right of the start key. In the touchscreen condition, participants began each trial by resting their finger on a start location at the bottom center of the screen. After the stimulus array appeared, participants responded by reaching to touch one of two response targets located toward the top corners of the screen.

Given that keyboards and touchscreen displays are relatively affordable and widely used interfaces, we sought to compare whether one interface provided substantially higher reliability estimates than the other. We expected that the touchscreen condition would produce better reliability estimates than the keyboard condition for two primary reasons. First, the touchscreen condition allows the participant to maintain fixation on the display rather than splitting their attention between two different spatial locations (i.e., the display and the keyboard in the keyboard condition). Second, we expected that the larger reaching movements required in the touchscreen condition would provide ample time for participants to correct errors, whereas the keyboard condition might encourage rapid responses and higher error rates, resulting in more variance associated with speed–accuracy trade-off effects.

## Participants

In light of our previous research with the flanker task, we aimed to have a minimum final sample size of 45 participants, and we set out to collect as many participants as possible within the course of an academic semester. A total of 53 participants ranging from 18 to 27 years of age participated in the study. The final sample consisted of 51 participants (*M* = 19.7 years, *SD* = 2.0) after two participants were removed due to generating an exceptionally high number of errors in the keyboard condition (> 15%, more than double the next highest error rate), despite being instructed to avoid making errors. Of the final sample, 36 participants identified as female, 13 as male, and two preferred not to respond. The final sample was ethnically diverse, with participants reporting as their primary ethnic group as follows: 21 Asian, 12 New Zealand European, three Pacific peoples, two Māori, one Middle Eastern, five Other, four Other European, and three preferred not to say. Five of the participants reported multiple ethnic backgrounds (one Asian and New Zealand European, one New Zealand European and Māori, one New Zealand European and Pacific peoples, and one New Zealand European and Other). All participants included in the final sample were between 18 and 27 years of age, had a normal or corrected-to-normal vision, were able to perform normal reaching movements, understood English, and had no previous diagnosis of a social or cognitive impairment. Three participants were left-handed, 46 were right-handed, and two preferred not to respond.

Participants were recruited through printed flyers, online postings, and the University of Auckland School of Psychology research participation pool. All participants received either a $15 NZD supermarket voucher or course credit for participating. All participants provided informed written consent and completed a demographics survey before the experiment. The protocol was approved by the University of Auckland Human Participants Ethics Committee (UAHPEC023445).

## Methods

Participants were presented with a two-alternative flanker task under two different response conditions: a keyboard condition and a touchscreen condition. All participants received both conditions, with the presentation order counterbalanced across participants. For each condition, participants initially completed a practice block of 12 trials, followed by four blocks of 48 trials. In both conditions, participants were seated in front of a keyboard and a touchscreen display (Dell P2418HT 24″ LCD Touchscreen Monitor), with the screen placed at an angle of approximately 60 degrees and the bottom edge of screen approximately 25 cm from the edge of the table. The screen displayed instructions on how to complete the task and reminded participants to go as fast as they could while trying to avoid making errors.

**Fig. 3:**
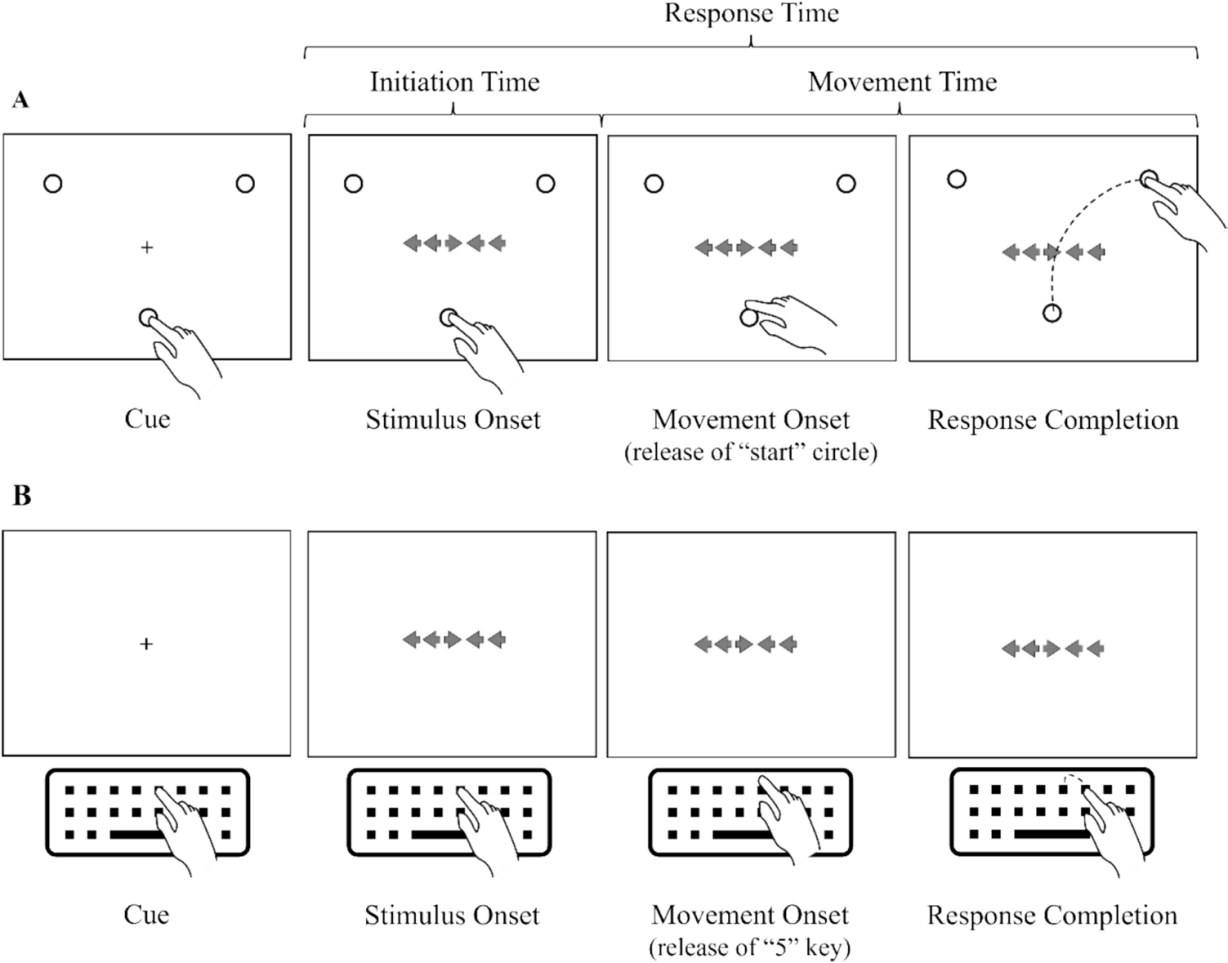
Illustration of touchscreen and keyboard conditions of the flanker task from Study 2. Note. A Illustration of the touch screen condition of the flanker task. B Illustration of the keyboard condition of the flanker task

In the keyboard condition, participants were required to press and hold their index finger on the number “5” key on the keyboard to begin each trial (see Fig. 3). In the touchscreen condition, participants were required to press and hold their index finger within an initiation circle (approximately 3 cm in diameter) that appeared on the bottom of the screen (approximately 6 cm from the bottom of the screen to the center of the circle) to begin each trial. Once participants had initiated a trial (by pressing their finger on the initiation circle or holding down the “5” key, as appropriate) a cue would be presented for 1 s, followed by the stimulus array. If the participant lifted their index finger before the fixation cross disappeared, the screen went blank, and the participant had to return their finger to the start location (key or circle) to restart the trial sequence.

The stimulus array was presented in the center of the screen in both the keyboard and touchscreen conditions and comprised five arrows (each arrow approximately 1.3 cm width × 2.5 cm height, and the entire stimulus array measuring approximately 6.5 cm width × 2.5 cm height), with the central arrow being the target stimulus. The arrows on either side of the central arrow (the flanking arrows) would point in a direction that was either congruent with the central arrow (e.g., < < < < <) or incongruent (e.g., < < > < <). The stimulus array was presented in the center of the display in both the keyboard condition and the touchscreen condition, with the center of the array located approximately 15 cm from the bottom and top edges of the display and 26.5 cm from the left and right edges of the display.

Participants were required to make a left or right response based on the direction of the target stimulus (the central arrow). In the keyboard condition, participants were instructed to press the “4” key for left responses or the “6” key for right responses. For the touchscreen condition, participants were presented with two response circles (one located approximately 4.5 cm from the top edge of the screen and 10 cm from the left edge of the screen, the other located approximately 4.5 cm from the top of the screen and 10 cm from the right edge of the screen) and were required to touch one of these circles to make their response. A high tone sounded for correct responses provided in the allotted time (600 Hz for 200 ms) and a low tone sounded for incorrect responses or responses that exceeded the allotted time (300 Hz for 200 ms). The allotted time for the stimulus onset period (which starts when the stimulus array is displayed and ends when a participant lifts their index finger from the starting key/circle) was 4 s, and then another 4 s for the movement onset period (which starts when a participant lifts their finger from the starting key/circle and ends when they press a response key/circle.

Participants were instructed to use their index finger throughout both conditions of the experiment to ensure they lifted their index finger from the relevant circle/key to initiate a trial and then moved that same finger to the appropriate circle/key and press down to make a response. This method allowed for the measurement of initiation time (IT; time elapsed between stimulus presentation and releasing the start key/circle), movement time (MT; time elapsed between IT and participants pressing a response key/circle), and response time (RT; the time elapsed between stimulus presentation and the pressing of a response key/circle).

## Results

The data, analysis scripts, task materials, and Supplementary Materials for Study 2 are available at https://osf.io/pvd7t/. Average error rates were low for both the keyboard (*M* = 1.2%, *SD* = 1.5%) and the touchscreen (*M* = 0.1%, *SD* = 0.7%) conditions. Before analyzing RT, IT, or MT, the first trial from each block was excluded, as were all trials featuring an error or trials following an error (Danielmeier & Ullsperger, 2011) and all trials featuring RTs under 200 ms or over 2.5 s. The mean and standard deviation of each participants’ overall response time on each condition were then calculated. Any trials featuring a RT greater than 3 SDs from an individual’s own mean RT for each condition were considered outliers and subsequently removed. Across all participants, an average of 3.6% of trials were removed for the keyboard condition (*SD* = 2.8) and 2.3% removed for the touchscreen condition (*SD* = 1.6). The descriptive statistics for average RT, IT, MT, and error rate are shown in Table S9 in Sect. 3 of the Supplementary Materials. As in Study 1, we applied log-transformations to the data and calculated congruency effects for RT, IT, and MT. Table 5 presents the descriptive statistics for the congruency effects observed in both the non-transformed and the log-transformed RT, IT, and MT data.

**Table 5:** Descriptive statistics for congruency effects observed in non-transformed and log-transformed response time, initiation time, and movement time for the keyboard and touchscreen flanker task conditions

Task	Measure	*M*	*SD*	Min	Max	Skew	Kurtosis
Keyboard	RT	85	33	40	217	2.27	6.9
	IT	30	36	−11	173	2.25	6.58
	MT	56	28	2	128	0.24	−0.36
	RT (Log-Transformed)	0.142	0.044	0.069	0.292	0.85	1.43
	IT (Log-Transformed)	0.058	0.065	−0.093	0.282	0.73	1.95
	MT (Log-Transformed)	0.254	0.129	0.007	0.54	0.12	−0.82
Touchscreen	RT	71	29	22	199	2.18	7.06
	IT	21	32	−9	177	2.88	9.91
	MT	50	23	−3	104	0.19	−0.34
	RT (Log-Transformed)	0.088	0.029	0.031	0.166	0.65	0.11
	IT (Log-Transformed)	0.037	0.05	−0.036	0.214	1.5	2.4
	MT (Log-Transformed)	0.127	0.066	−0.013	0.309	0.62	0.2

To examine the correlation between the component scores that were used to generate congruency effects, we correlated average performance on congruent trials with average performance on incongruent trials for each dependent measure for each participant. Table 10 of the Appendix presents these correlations for each condition. These correlations were uniformly high, ranging from 0.91 to 0.99.

Next, we calculated component reliabilities for congruent and incongruent trials separately, as well as reliabilities for the congruency effect. All datasets were analyzed using the permutation-based split-half approach used in Study 1 (Parsons, 2021), with 5000 random splits for each estimate. The Spearman–Brown corrected reliability estimates for the component scores observed in each condition are presented in Table 11 of the Appendix. The estimates for the component scores were uniformly high across all measures and across both conditions, ranging from 0.98 to 1.00.

**Fig. 4:**
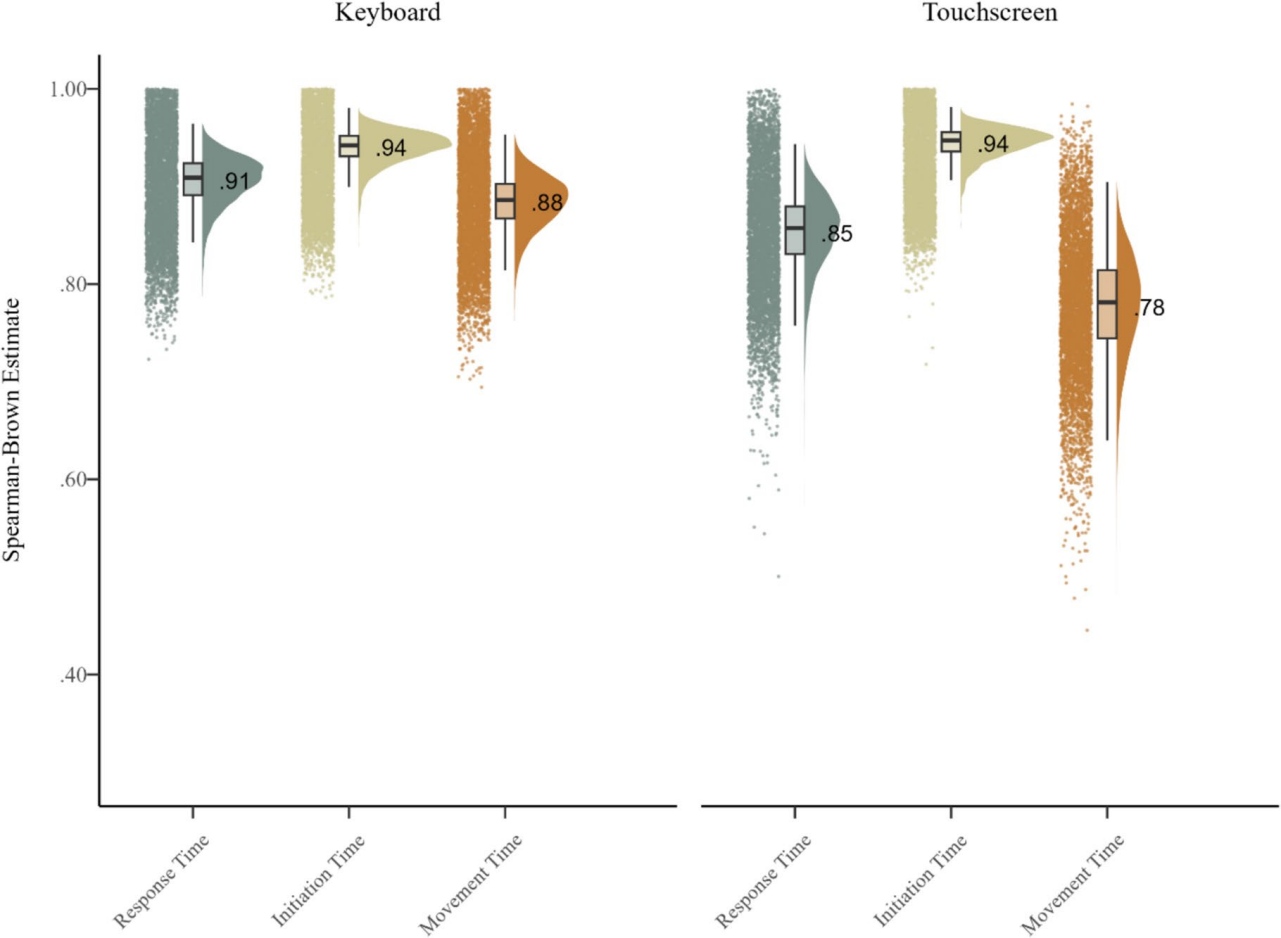
Raincloud plot for the difference score (congruency effect) reliability estimates observed in the non-transformed data from the keyboard and touchscreen conditions for the flanker task

Figure 4 presents the reliability estimates of the congruency effects observed in each dependent measure and condition. The results revealed acceptable to excellent reliability estimates for all measures across both the keyboard and touchscreen conditions. The keyboard condition generated estimates of 0.91 in RT, 0.94 in IT, and 0.88 in MT, while the touchscreen condition generated estimates of 0.85 in RT, 0.94 in IT, and 0.78 in MT. Examination of the log-transformed data revealed a similar pattern of results, with the keyboard condition generating estimates of 0.88 in RT, 0.87 in IT, and 0.90 in MT, while the touchscreen condition generated estimates of 0.81 in RT, 0.83 in IT, and 0.85 in MT (see Fig. 6 of the Appendix). The correlations between each of the computed congruency effects are also presented in Sect. 3 of the Supplementary Materials.

### Exploratory analyses

**Fig. 5:**
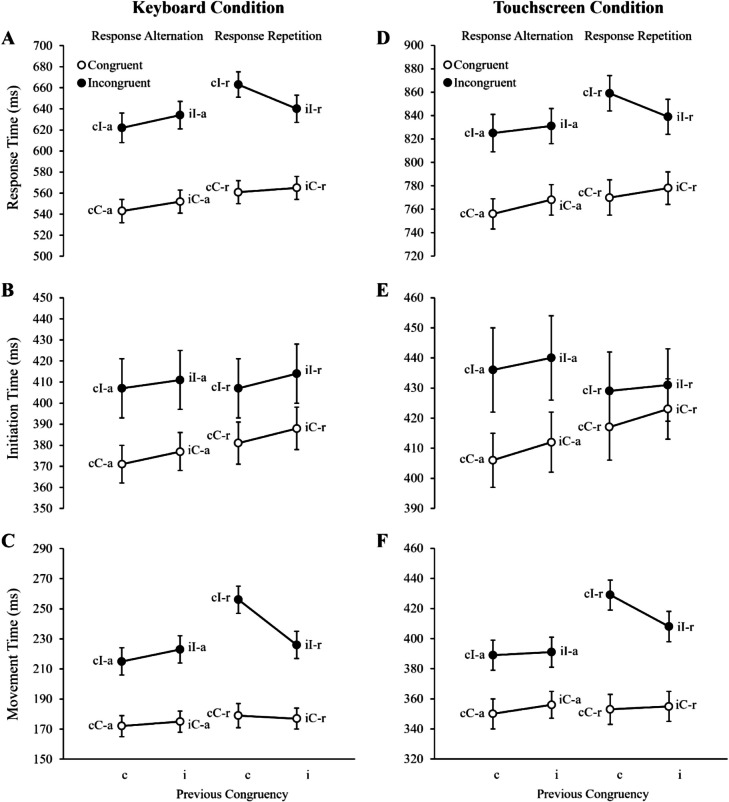
Response time, initiation time, and movement time results for the keyboard condition and touchscreen condition. *Note.* Average response times, initiation times, and movement times from the keyboard condition (**A**–**C**) and touchscreen condition (**D**–**F**) as a function of trial *n* congruency (**C** vs. **I**), trial *n*−1 congruency (**c** vs. **i**), and response repetition type (response alternation vs. response repetition). *Error bars* show standard errors

**Fig. 6:**
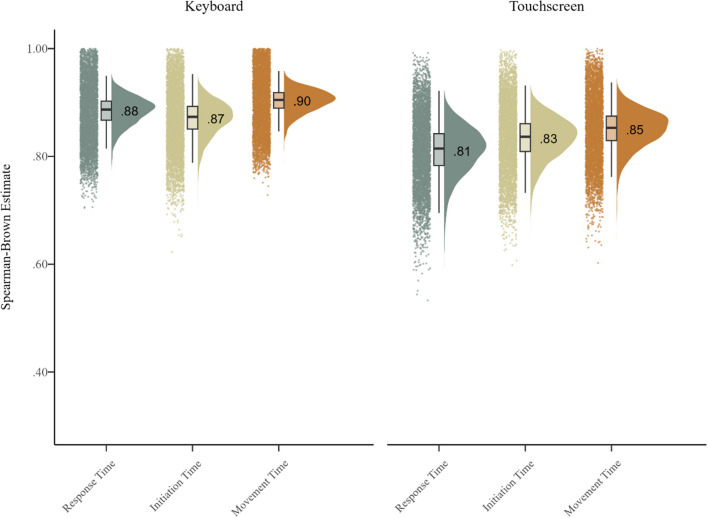
Raincloud plot for the difference score (congruency effect) reliability estimates observed in the log-transformed data from the keyboard and touchscreen conditions for the flanker task featured in Study 2

Although the current study was designed to evaluate the reliability of press-and-release flanker tasks, we also evaluated the sequence effects observed in RT, IT, and MT across both conditions via a series of 2 (Trial *n* congruency: C vs. I) × 2 (Trial *n*−1 congruency: c vs. i) × 2 (Response repetition type: alternation vs. repetition) ANOVAs. As discussed in the Introduction, previous reach-tracking research indicates that the pattern of RT effects commonly observed in button-press versions of the flanker task reflect distinct patterns of effects in IT and MT (Erb & Marcovitch, 2018; Erb et al., 2021a, b). Consequently, it is important to confirm whether the tasks used in Study 2 capture distinct patterns of effects in IT and MT, as researchers may want to weigh each task’s ability to dissociate IT and MT effects against the task’s reliability. We briefly summarize the results of these analyses below. For the full results of these analyses, see Sect. 4 of the Supplementary Materials. Average performance across both conditions is displayed in Fig. 5 for each dependent measure.

Consistent with previous reach-tracking investigations of the flanker task (e.g., Erb & Marcovitch, 2018), RTs revealed significant three-way interactions in both the keyboard condition, *F*(1, 50) = 25.02, *p* < 0.001, and the touchscreen condition, *F*(1, 50) = 6.68, *p* = 0.013. The pattern observed in RTs reflected distinct sequence effects in ITs and MTs. ITs revealed main effects of trial *n* congruency in the keyboard condition, *F*(1, 50) = 35.64, *p* < 0.001, and the touchscreen condition, *F*(1, 50) = 24.47, *p* < 0.001, as well as main effects of trial *n*−1 congruency in the keyboard condition, *F*(1, 50) = 13.34, *p* < 0.001, and the touchscreen condition, *F*(1, 50) = 7.20, *p* = 0.010. In contrast, MTs revealed significant three-way interactions in both the keyboard condition, *F*(1, 50) = 21.43, *p* < 0.001, and the touchscreen condition, *F*(1, 50) = 6.35, *p* = 0.015, with this interaction driven by particularly slow MTs on incongruent trials that were preceded by a congruent trial and that featured a response repetition (cI-r trials).

### Discussion: Study 2

The results of Study 2 provided strong evidence that release-and-press response methods can generate high congruency effect reliability estimates in the Eriksen flanker task, one of the most widely used measures of conflict processing in the psychological and brain sciences (Erb et al., 2021a, b; MacLeod et al., 2010; Zelazo et al., 2014). The keyboard condition of the release-and-press flanker task generated congruency effect reliability estimates of 0.91 in RT, 0.94 in IT, and 0.88 in MT, whereas the touchscreen condition generated estimates of 0.85 in RT, 0.94 in IT, and 0.78 in MT. Importantly, error rates in both conditions of the task were low, though errors were more common in the keyboard task (1.2%) than the touchscreen task (0.1%). Again, it is important to note that the reliability estimates observed in Study 2 are unlikely to have stabilized given that the sample size was relatively small (Schönbrodt & Perugini, 2013).

The results did not support our expectation that the touchscreen task would provide more reliable congruency effect estimates than the keyboard task, though errors were more common in the keyboard task, as anticipated. Given that the results of Study 1 suggested that CURV provides a more reliable measure of the congruency effect than MT in adults performing reach-tracking flanker tasks, it is possible that MTs in the touchscreen task in Study 2 reflected greater variations in movement speed than those of the keyboard task. On this view, larger reach distances in the touchscreen task provided more opportunities for participants to correct incorrect responses, as well as more variability in how rapidly these corrections were performed.

In tasks measuring the spatial characteristics of movement trajectories, such variations in movement speed might be of less concern, assuming that the spatial measures reliably assess individual differences in the extent to which participants approached the incorrect response. However, in the absence of such spatial measures, it may be advisable to reduce the reach distance to minimize the impact of variations in the speed of corrective movements. It is therefore possible that the reliability of the touchscreen task used in the current study could be enhanced by decreasing the distance between the start location and the responses. That said, the keyboard task provided excellent reliability estimates in the current study and is likely easier for researchers to incorporate into their current procedures.

Finally, we also explored the extent to which the keyboard and touchscreen conditions captured the same sequence effects observed in previous reaching tasks (e.g., Erb & Marcovitch, 2018; Smith et al., 2022). Both conditions captured the same general effects previously reported in RT, IT, and MT, indicating that expensive reach-tracking equipment and custom-built response boxes are not necessary to target dissociable sequence effects behaviorally. Additionally, the keyboard condition consistently generated more robust effects than the touchscreen condition, suggesting that a standard keyboard may be all that is required to generate robust experimental effects and reliable individual difference measures in the flanker task.

## General discussion

Longstanding questions regarding the reliability of experimental tasks targeting conflict processing have attracted renewed interest in recent years (Draheim et al., 2019; Enkavi et al., 2019; Hedge et al., 2018), leading some to question whether the field is experiencing a reliability crisis (Rouder & Mehrvarz, 2023). To address these concerns, researchers have sought to develop more reliable versions of widely used congruency tasks such as the flanker, Stroop, and Simon tasks (Burgoyne, 2023; Draheim et al., 2021; Kucina et al., 2023). The current study investigated whether the reliability of congruency tasks can also be enhanced with reaching methods that provide participants more opportunities to detect and override incorrect responses.

The results of Study 1 demonstrate that reaching methods provide a promising approach to enhancing the reliability of tasks commonly believed to reflect differences in conflict processing. Reach-tracking versions of the flanker and Stroop tasks routinely generated acceptable to excellent congruency effect reliability estimates, with RT estimates generally performing as well or better than those observed in IT, MT, and CURV. Adult performance of the flanker task generated higher congruency effect reliability estimates in CURV than MT in each of the datasets evaluated in Study 1, whereas child performance of the flanker task did not reveal this trend. Congruency effect reliability estimates in the Simon task were relatively low and showed pronounced variability across measures. CURV provided the highest reliability estimates for the Simon congruency effect observed in 6- to 8-year-olds (0.84) and 10- to 12-year-olds (0.57), while the adult performance revealed reliability estimates of 0.72 in RT, 0.76 in IT, and 0.75 in CURV.

Study 1 did not provide strong evidence that the reliability of RT congruency effects in reaching tasks is impaired due to the measure reflecting the contribution of multiple subprocesses underlying performance. However, IT did provide descriptively higher reliability estimates than RT in all eight of the adult datasets evaluated in Study 1 and descriptively higher reliability estimates than MT in seven out of the eight adult datasets evaluated in Study 1. While acknowledging that these descriptive differences were often quite minimal, the general trend observed in Study 1 suggests that IT might provide a particularly reliable temporal measure of congruency effects in adults.

Although the results of Study 1 presented compelling evidence that the reliability of congruency tasks can be enhanced by replacing standard button-press tasks with reaching tasks, many of the techniques used to record three-dimensional hand movements are expensive and difficult to use, limiting their accessibility (Erb, 2018). In light of the promising reliability estimates generated by a release-and-press version of the flanker task from Smith et al. (2022), Study 2 investigated the extent to which the same approach could be used to enhance reliability when measuring performance with a standard keyboard or a touchscreen display. The results of Study 2 indicated that release-and-press tasks generate low error rates and excellent congruency effect reliability estimates, particularly in IT, with estimates of 0.94 in the keyboard task and 0.94 in the touchscreen task. Notably, RT estimates were above 0.80 in each condition, with the keyboard task generating a congruency effect reliability estimate of 0.91 in RT and the touchscreen task generating an estimate of 0.85.

The results of Study 2 lend credence to the notion that reaching tasks generate high-reliability estimates by reducing the contributions of speed–accuracy trade-off effects on performance relative to button-press tasks. Traditional button-press tasks encourage a more ballistic form of responding, making it relatively difficult for participants to detect and override an incorrect response. This results in more frequent errors, particularly when speed is emphasized, and introduces increased variance associated with individual differences in strategy (Hedge et al., 2022), as well as substantial variance associated with responses leading up to or following an error (e.g., pre-error speeding and post-error slowing; Pfister & Foerster, 2022).

In reaching tasks, small increases in response speed are likely to incur a correction cost (e.g., in the form of a large movement curvature or slower movement time) rather than resulting in an error. The size of these correction costs likely reflects the prepotency of the initial response, such that faster movement initiations will be offset to some extent by larger movement trajectories or longer movement times. This general trade-off was observed in Study 2 in the form of strong negative correlations between the size of the congruency effects observed in IT and MT, with smaller average IT congruency effects correlating with larger average MT congruency effects (see Table S10 of the Supplementary Materials). Although the results of Study 2 lend credence to the notion that reaching tasks reduce the contributions of speed–accuracy trade-off effects on performance, future research is needed to test this possibility. This could be accomplished, for example, by altering the instructions provided to participants to emphasize either speed or accuracy.

Reaching tasks may also provide enhanced reliability estimates relative to traditional button-press tasks because reaching tasks are better suited to capturing correction costs. Although error rates tend to be higher in button-press tasks than reaching tasks, electromyography measurements indicate that participants frequently generate competing response activations in button-press tasks (Burle et al., 2014; Coles et al., 1985; Eriksen et al., 1985), suggesting that participants do detect and override incorrect responses in button-press tasks. In such cases, the costs of correcting an incorrect response are relatively minimal compared to reaching tasks, as participants can rapidly press the correct button upon determining that their initial response is incorrect. By contrast, the costs associated with correcting an incorrect response are likely to be more pronounced in reaching tasks because any movement toward the incorrect response must be compensated for by a larger movement toward the correct response.

Although future research is needed to identify the extent to which the release-and-press method can be used to generate highly reliable estimates of performance across different tasks and groups of participants, the results of Study 2 have direct implications for researchers investigating a wide range of topics beyond conflict processing. Button-press measures of RT and accuracy have long served as cornerstones of perceptual and cognitive psychology. As noted by Draheim et al. (2019), RT and accuracy difference scores are ubiquitous throughout the psychological sciences, including perceptual, cognitive, clinical, and social psychology. Our findings indicate that researchers targeting individual or group differences across these sub-fields of psychology could potentially increase the reliability of their assessments by simply replacing standard, ballistic button-press response methods with a release-and-press response method without needing to purchase additional equipment.

The methods used in Study 2 are also well suited for research outside of standard laboratory settings. In contrast to reach-tracking techniques requiring an electromagnetic position and orientation recording system or an array of high-speed cameras, keyboard- and touchscreen-based tasks can be administered in classroom, museum, or clinical settings with ease. Such tasks are also well suited for online data collection. Notably, the tasks used in Study 2 were developed to work with Pavlovia for online data collection and have been made available at the OSF website corresponding to this article.

A critical question raised by the current study concerns whether alternative hand-tracking approaches not explored in the present study also produce reliability metrics as high as those reported here. Mouse tracking, for instance, has emerged as a valuable hand-tracking methodology in cognitive research in recent years (Freeman & Ambady, 2010; Hehman et al., 2015; Kieslich et al., 2019; Scherbaum & Dshemuchadse, 2020), providing an affordable and accessible alternative to electromagnetic reach-tracking systems. Recent work from Unsworth and Miller (2024) indicates that mouse-tracking congruency tasks tend to generate congruency effect reliability estimates around 0.70. For example, their first study featured data from over 210 adult participants who performed both a mouse-tracking version and a standard button-press version of the Stroop task. The authors found that the Stroop effect observed in their area under the curve (AUC) measure exhibited a split-half reliability of 0.72, whereas the Stroop effect observed in RT in the button-press task was 0.52.

In a second study featuring data from more than 240 adult participants, Unsworth and Miller (2024) reported similar split-half estimates for the Stroop effects observed in AUC in a mouse-tracking task (0.67) and in RT in a button-press task (0.60). Similar split-half estimates were also reported for the congruency effects observed in AUC for a mouse-tracking version of the flanker task (0.66) and in a button-press flanker task (0.69). Thus, the existing data suggest that reaching versions of the flanker and Stroop tasks may produce more reliable congruency effect estimates in adults than mouse-tracking versions of the tasks. However, a direct comparison of both versions of the tasks is needed. It is also important to note that there is considerable variability in how familiar participants are with using a mouse, particularly for younger age groups (Lane & Ziviani, 2010), compared to more universally familiar embodied reaching movements. Consequently, additional research is needed to explore the extent to which mouse-tracking measures generate high-reliability estimates across different age groups.

In addition to concerns raised about the reliability of congruency tasks, researchers have also questioned the validity of the tasks. Congruency tasks are commonly suggested to measure *executive attention* or *attention control*, but the congruency effects observed in tasks often fail to correlate strongly (for a review, see Rouder & Haaf, 2019). This has led some researchers to reasonably question whether attention control lacks coherence as a unified construct (Hedge et al., 2022; Rey-Mermet et al., 2018; Rouder & Haaf, 2019), while others have suggested that attention control does emerge as a coherent construct when more reliable tasks are used (Draheim et al., 2021). Although the current study specifically focused on the issue of reliability, an important limitation to acknowledge is that the study was not designed to assess the validity of the reported reach-tracking measures. Unsworth and Miller (2024) evaluated the validity of mouse-tracking versions of the Stroop and flanker tasks by collecting a range of other measures related to attention control and working memory. Their results indicated that AUC on incongruent trials in the Stroop task and MT on incongruent trials in the flanker task were related to attention control. In future work, we plan to build on these findings by collecting multiple measures of attention control and working memory alongside measures of release-and-press congruency tasks.

## Conclusion

The results of Study 1 presented important implications for research exploring developmental and individual differences in conflict processing, demonstrating that reach-tracking flanker and Stroop tasks routinely generate excellent reliability metrics. Study 2 built on these findings by demonstrating that release-and-press versions of the flanker task implemented using a standard keyboard or a touchscreen interface can generate reliability estimates ranging from acceptable to excellent while also capturing distinct sequence effects in IT and MT. Thus, we believe that behavior research methods allowing for continuous – as opposed to ballistic – manual responses present another promising approach to enhancing the reliability of congruency tasks that can complement other recent approaches (e.g., Burgoyne et al., 2023; Kucina et al., 2023). Although other approaches present important strengths that are well suited for many research needs, such as brief administration times (e.g., Burgoyne et al., 2023), hand-tracking and release-and-press methods present a promising approach to reconciling experimental and differential research domains, helping to bridge a longstanding divide within the study of psychology (Cronbach, 1957). By providing more robust measures of experimental effects, these methods offer a framework for gaining insights into the cognitive dynamics underlying attention and control, as well as more reliable measures for investigating developmental and individual differences.

## Electronic supplementary material

Below is the link to the electronic supplementary material.

Supplementary file1 (DOCX 244 KB)

## Data Availability

The data, analysis scripts, task materials, and supplementary materials for this project are available at https://osf.io/pvd7t/.

## Appendix

See Tables 6, 7, 8, 9, 10, 11 and Fig. 6

**Table 6:** Component score (congruent vs incongruent) correlations for response time, initiation time, movement time and curvature for flanker task datasets featured in Study 1

Experiment	Age Group	Measure	*r*	*p*	95% confidence interval	
					Lower	Upper
Erb et al., (2016, Exp. 2)	Young adults	RT	0.99	< 0.001	0.99	1
		IT	0.99	< 0.001	0.99	1
		MT	0.98	< 0.001	0.96	0.99
		CURV	0.76	< 0.001	0.59	0.87
Erb et al., (2018, Exp. 1)	5- to 10-year-olds	RT	0.92	< 0.001	0.87	0.95
		IT	0.91	< 0.001	0.85	0.94
		MT	0.86	< 0.001	0.77	0.91
		CURV	0.67	< 0.001	0.51	0.79
Erb et al., (2018, Exp. 2)	Young adults	RT	0.98	< 0.001	0.95	0.99
		IT	0.96	< 0.001	0.90	0.98
		MT	0.98	< 0.001	0.96	0.99
		CURV	0.76	< 0.001	0.52	0.89
Erb and Marcovitch (2018)	6- to 8-year-olds	RT	0.75	< 0.001	0.59	0.86
		IT	0.78	< 0.001	0.63	0.87
		MT	0.61	< 0.001	0.39	0.77
		CURV	0.37	= 0.01	0.09	0.60
	10- to 12-year-olds	RT	0.95	< 0.001	0.91	0.97
		IT	0.97	< 0.001	0.95	0.98
		MT	0.84	< 0.001	0.73	0.91
		CURV	0.60	< 0.001	0.38	0.76
	Young adults	RT	0.94	< 0.001	0.89	0.97
		IT	0.93	< 0.001	0.88	0.96
		MT	0.96	< 0.001	0.93	0.98
		CURV	0.60	< 0.001	0.37	0.76
Erb et al. (2020)	Older adults	RT	0.89	< 0.001	0.81	0.94
		IT	0.92	< 0.001	0.86	0.96
		MT	0.81	< 0.001	0.68	0.89
		CURV	0.64	< 0.001	0.42	0.78
Erb et al., (2021a, b)	Young adults	RT	0.97	< 0.001	0.96	0.98
		IT	0.97	< 0.001	0.95	0.98
		MT	0.96	< 0.001	0.95	0.97
		CURV	0.55	< 0.001	0.42	0.66
Smith et al. (2022)	6- to 8-year-olds	RT	0.79	< 0.001	0.65	0.88
		IT	0.83	< 0.001	0.71	0.91
		MT	0.80	< 0.001	0.66	0.88
	Young adults	RT	0.96	< 0.001	0.93	0.98
		IT	0.98	< 0.001	0.96	0.99
		MT	0.94	< 0.001	0.89	0.97

**Table 7:** Component score (congruent vs incongruent) correlations for response time, initiation time, movement time and curvature for Stroop and Simon task datasets featured in Study 1

Experiment	Task	Age group	Measure	*r*	*p*	95% confidence interval	
						Lower	Upper
Erb et al., (2016, Exp. 1)	Stroop	Young adults	RT	0.95	< 0.001	0.89	0.98
			IT	0.96	< 0.001	0.92	0.98
			MT	0.95	< 0.001	0.88	0.98
			CURV	0.71	< 0.001	0.42	0.86
Erb and Marcovitch (2019)	Simon	6- to 8-year-olds	RT	0.95	< 0.001	0.90	0.97
			IT	0.97	< 0.001	0.94	0.98
			MT	0.82	< 0.001	0.67	0.90
			CURV	0.39	= 0.02	0.07	0.63
		10- to 12-year-olds	RT	0.94	< 0.001	0.89	0.97
			IT	0.96	< 0.001	0.92	0.98
			MT	0.95	< 0.001	0.91	0.98
			CURV	0.79	< 0.001	0.62	0.89
		Young adults	RT	0.98	< 0.001	0.96	0.99
			IT	0.98	< 0.001	0.96	0.99
			MT	0.98	< 0.001	0.96	0.99
			CURV	0.68	< 0.001	0.46	0.83

**Table 8:** Spearman–Brown corrected split-half reliabilities of the congruent and incongruent component scores for response time, initiation time, movement time and curvature for flanker task datasets featured in Study 1

Experiment	Age group	Measure	Congruent			Incongruent		
			*r*	95% confidence interval		*r*	95% confidence interval	
				Lower	Upper		Lower	Upper
Erb et al., (2016, Exp. 2)	Young adults	RT	1.00	0.99	1.00	1.00	0.99	1.00
		IT	0.99	0.99	1.00	1.00	0.99	1.00
		MT	0.99	0.99	1.00	0.99	0.98	0.99
		CURV	0.92	0.86	0.95	0.90	0.84	0.94
Erb et al., (2018, Exp. 1)	5- to 10-year-olds	RT	0.97	0.95	0.99	0.98	0.97	0.99
		IT	0.96	0.93	0.98	0.97	0.94	0.98
		MT	0.94	0.87	0.98	0.97	0.93	0.99
		CURV	0.61	0.37	0.77	0.76	0.59	0.85
Erb et al., (2018, Exp. 2)	Young adults	RT	0.99	0.99	1.00	0.99	0.99	1.00
		IT	0.99	0.97	1.00	0.99	0.99	1.00
		MT	1.00	0.99	1.00	0.99	0.99	1.00
		CURV	0.96	0.93	0.98	0.97	0.95	0.99
Erb and Marcovitch (2018)	6- to 8-year-olds	RT	0.97	0.95	0.98	0.98	0.96	0.99
		IT	0.96	0.94	0.98	0.97	0.94	0.99
		MT	0.93	0.90	0.96	0.93	0.88	0.97
		CURV	0.74	0.60	0.84	0.88	0.82	0.93
	10- to 12-year-olds	RT	0.99	0.98	0.99	0.98	0.98	0.99
		IT	0.99	0.98	0.99	0.98	0.97	0.99
		MT	0.98	0.97	0.99	0.93	0.89	0.96
		CURV	0.78	0.67	0.87	0.85	0.76	0.91
	Young adults	RT	0.99	0.98	0.99	0.99	0.97	0.99
		IT	0.99	0.98	0.99	0.98	0.96	0.99
		MT	0.99	0.99	1.00	0.99	0.98	0.99
		CURV	0.93	0.89	0.96	0.94	0.90	0.96
Erb et al. (2020)	Older adults	RT	0.99	0.98	0.99	0.98	0.96	0.99
		IT	0.99	0.98	0.99	0.98	0.96	0.99
		MT	0.95	0.92	0.97	0.93	0.88	0.96
		CURV	0.94	0.90	0.96	0.96	0.94	0.98
Erb et al., (2021a, b)	Young adults	RT	0.99	0.99	1.00	0.99	0.99	1.00
		IT	0.99	0.99	0.99	0.99	0.99	0.99
		MT	0.99	0.99	1.00	0.99	0.99	0.99
		CURV	0.95	0.93	0.96	0.96	0.95	0.97
Smith et al. (2022)	6- to 8-year-olds	RT	0.96	0.93	0.98	0.98	0.96	0.99
		IT	0.96	0.93	0.98	0.97	0.95	0.98
		MT	0.94	0.91	0.97	0.98	0.95	0.99
	Young adults	RT	0.99	0.98	0.99	0.98	0.97	0.99
		IT	0.99	0.98	0.99	0.99	0.98	0.99
		MT	0.99	0.98	0.99	0.97	0.96	0.99

**Table 9:** Spearman–Brown corrected split-half reliabilities of the congruent and incongruent component scores for response time, initiation time, movement time and curvature for Stroop and Simon datasets featured in Study 1

Experiment	Task	Age group	Measure	Congruent			Incongruent		
				*r*	95% confidence interval		*r*	95% confidence interval	
					Lower	Upper		Lower	Upper
Erb et al., (2016, Exp. 1)	Stroop	Young adults	RT	0.99	0.99	1.00	0.99	0.99	1.00
			IT	0.99	0.98	1.00	0.99	0.99	1.00
			MT	0.99	0.98	1.00	0.99	0.98	1.00
			CURV	0.95	0.90	0.98	0.91	0.85	0.96
Erb and Marcovitch (2019)	Simon	6- to 8-year-olds	RT	0.97	0.94	0.98	0.98	0.96	0.99
			IT	0.96	0.93	0.98	0.97	0.95	0.98
			MT	0.93	0.87	0.96	0.94	0.90	0.97
			CURV	0.72	0.56	0.84	0.91	0.87	0.95
		10- to 12-year-olds	RT	0.97	0.95	0.98	0.97	0.96	0.98
			IT	0.96	0.94	0.98	0.97	0.95	0.98
			MT	0.98	0.96	0.99	0.98	0.96	0.99
			CURV	0.88	0.81	0.93	0.90	0.84	0.95
		Young adults	RT	0.99	0.98	1.00	0.99	0.99	1.00
			IT	0.99	0.99	1.00	0.99	0.99	1.00
			MT	0.99	0.98	0.99	0.99	0.98	0.99
			CURV	0.89	0.80	0.94	0.92	0.88	0.96

**Table 10:** Component score (congruent vs incongruent) correlations for response time, initiation time, and movement time for the keyboard and touchscreen flanker task conditions featured in Study 2

Experiment	Measure	*r*	*p*	95% confidence interval	
				Lower	Upper
Keyboard	RT	0.94	< 0.001	0.89	0.96
	IT	0.98	< 0.001	0.97	0.99
	MT	0.91	< 0.001	0.84	0.95
Touchscreen	RT	0.97	< 0.001	0.94	0.98
	IT	0.99	< 0.001	0.97	0.99
	MT	0.95	< 0.001	0.91	0.97

**Table 11:** Spearman–Brown corrected split-half reliabilities of the congruent and incongruent component scores for response time, initiation time, and movement time for the keyboard and touchscreen flanker task conditions featured in study 2

Experiment	Congruency	Measure	*r*	95% confidence interval	
				Lower	Upper
Keyboard	Congruent	RT	0.99	0.99	1.00
		IT	0.99	0.99	1.00
		MT	0.99	0.99	0.99
	Incongruent	RT	0.99	0.99	1.00
		IT	0.99	0.99	1.00
		MT	0.98	0.98	0.99
Touchscreen	Congruent	RT	0.99	0.99	1.00
		IT	1.00	0.99	1.00
		MT	0.99	0.99	0.99
	Incongruent	RT	0.99	0.99	1.00
		IT	1.00	0.99	1.00
		MT	0.98	0.98	0.99

## Declarations

### Author Note

The data, analysis scripts, task materials, and supplementary materials for this project are available at https://osf.io/pvd7t/.

This manuscript presents a re-analysis of previously published data in Study 1 and analysis of original experimental data in Study 2. The analyses presented in this manuscript were not pre-registered. The R scripts used to perform the analyses reported in this manuscript are available at https://osf.io/pvd7t/.

## References

[CR1] Burgoyne, A. P., Tsukahara, J. S., Mashburn, C. A., Pak, R., & Engle, R. W. (2023). Nature and measurement of attention control. *Journal of Experimental Psychology: General,**152*(8), 2369–2402. 37079831 10.1037/xge0001408

[CR2] Burle, B., Spieser, L., Servant, M., & Hasbroucq, T. (2014). Distributional reaction time properties in the Eriksen task: Marked differences or hidden similarities with the Simon task? *Psychonomic Bulletin & Review,**21*, 1003–1010. 24302468 10.3758/s13423-013-0561-6 PMC4104006

[CR3] Coles, M. G., Gratton, G., Bashore, T. R., Eriksen, C. W., & Donchin, E. (1985). A psychophysiological investigation of the continuous flow model of human information processing. *Journal of Experimental Psychology: Human Perception and Performance,**11*(5), 529–553. 2932529 10.1037//0096-1523.11.5.529

[CR4] Creyos (n.d.) *Double trouble*. https://creyos.com/online-cognitive-tasks/double-trouble-test

[CR5] Cronbach, L. J. (1957). The two disciplines of scientific psychology. *The American Psychologist,**12*(11), 671–684. 10.1037/h0043943

[CR6] Danielmeier, C., & Ullsperger, M. (2011). Post-error adjustments. *Frontiers in Psychology,**2*, 233. 21954390 10.3389/fpsyg.2011.00233 PMC3173829

[CR7] Draheim, C., Mashburn, C. A., Martin, J. D., & Engle, R. W. (2019). Reaction time in differential and developmental research: A review and commentary on the problems and alternatives. *Psychological Bulletin,**145*(5), 508–535. 30896187 10.1037/bul0000192

[CR8] Draheim, C., Tsukahara, J. S., Martin, J. D., Mashburn, C. A., & Engle, R. W. (2021). A toolbox approach to improving the measurement of attention control. *Journal of Experimental Psychology: General,**150*(2), 242–275. 32700925 10.1037/xge0000783

[CR9] Erb, C. D. (2018). The developing mind in action: Measuring manual dynamics in childhood. *Journal of Cognition and Development, 19*(3), 233–247.

[CR10] Erb, C. D., Germine, L., & Hartshorne, J. K. (2023). Cognitive control across the lifespan: Congruency effects reveal divergent developmental trajectories. *Journal of Experimental Psychology. General,**152*(11), 3285–3291. 10.1037/xge0001429 37289513 10.1037/xge0001429

[CR11] Erb, C. D., & Marcovitch, S. (2018). Deconstructing the Gratton effect: Targeting dissociable trial sequence effects in children, pre-adolescents, and adults. *Cognition,**179*, 150–162. 29944979 10.1016/j.cognition.2018.06.007

[CR12] Erb, C. D., & Marcovitch, S. (2019). Tracking the within-trial, cross-trial, and developmental dynamics of cognitive control: Evidence from the Simon task. *Child Development,**90*(6), e831–e848. 29959776 10.1111/cdev.13111

[CR13] Erb, C. D., Moher, J., Sobel, D. M., & Song, J. H. (2016). Reach tracking reveals dissociable processes underlying cognitive control. *Cognition,**152*, 114–126. 27045465 10.1016/j.cognition.2016.03.015 PMC4868089

[CR14] Erb, C. D., Moher, J., Song, J. H., & Sobel, D. M. (2018). Reach tracking reveals dissociable processes underlying inhibitory control in 5- to 10-year-olds and adults. *Developmental Science,**21*(2), Article e12523. 10.1111/desc.12523 PMC6204061 28233397

[CR15] Erb, C. D., Smith, K. A., & Moher, J. (2021a). Tracking continuities in the flanker task: From continuous flow to movement trajectories. *Attention, Perception, & Psychophysics,**83*, 731–747. 10.3758/s13414-020-02154-4 33089369

[CR16] Erb, C. D., Touron, D. R., & Marcovitch, S. (2020). Tracking the dynamics of global and competitive inhibition in early and late adulthood: Evidence from the flanker task. *Psychology and Aging,**35*(5), 729. 32744854 10.1037/pag0000435

[CR17] Erb, C. D., Welhaf, M. S., Smeekens, B. A., Moreau, D., Kane, M. J., & Marcovitch, S. (2021b). Linking the dynamics of cognitive control to individual differences in working memory capacity: Evidence from reaching behavior. *Journal of Experimental Psychology: Learning, Memory, and Cognition,**47*(9), 1383–1402. 34197169 10.1037/xlm0001018

[CR18] Eriksen, B. A., & Eriksen, C. W. (1974). Effects of noise letters upon the identification of a target letter in a nonsearch task. *Perception & Psychophysics,**16*(1), 143–149. 10.3758/BF03203267

[CR19] Eriksen, C. W., Coles, M. G., Morris, L. R., & O’Hara, W. P. (1985). An electromyographic examination of response competition. *Bulletin of the Psychonomic Society,**23*(3), 165–168.

[CR20] Enkavi, A. Z., Eisenberg, I. W., Bissett, P. G., Mazza, G. L., MacKinnon, D. P., Marsch, L. A., & Poldrack, R. A. (2019). Large-scale analysis of test–retest reliabilities of self-regulation measures. *Proceedings of the National Academy of Sciences,**116*(12), 5472–5477. 10.1073/pnas.1818430116 PMC6431228 30842284

[CR21] Freeman, J. B., & Ambady, N. (2010). MouseTracker: Software for studying real-time mental processing using a computer mouse-tracking method. *Behavior Research Methods,**42*(1), 226–241. 20160302 10.3758/BRM.42.1.226

[CR22] Gratton, G., Coles, M. G., & Donchin, E. (1992). Optimizing the use of information: Strategic control of activation of responses. *Journal of Experimental Psychology: General,**121*(4), 480. 1431740 10.1037//0096-3445.121.4.480

[CR23] Hedge, C., Powell, G., Bompas, A., & Sumner, P. (2022). Strategy and processing speed eclipse individual differences in control ability in conflict tasks. *Journal of Experimental Psychology: Learning, Memory, and Cognition,**48*(10), 1448. 34591554 10.1037/xlm0001028 PMC9899369

[CR24] Hedge, C., Powell, G., & Sumner, P. (2018). The reliability paradox: Why robust cognitive tasks do not produce reliable individual differences. *Behavior Research Methods,**50*, 1166–1186. 28726177 10.3758/s13428-017-0935-1 PMC5990556

[CR25] Hehman, E., Stolier, R. M., & Freeman, J. B. (2015). Advanced mouse-tracking analytic techniques for enhancing psychological science. *Group Processes & Intergroup Relations,**18*(3), 384–401.

[CR26] Henson, R. K. (2001). Understanding internal consistency reliability estimates: A conceptual primer on coefficient alpha. *Measurement and Evaluation in Counseling and Development,**34*(3), 177–189.

[CR27] Incera, S., & McLennan, C. T. (2016). Mouse tracking reveals that bilinguals behave like experts. *Bilingualism: Language and Cognition*, *19*(3), 610–620.

[CR28] Kieslich, P. J., Henninger, F., Wulff, D. U., Haslbeck, J. M., & Schulte-Mecklenbeck, M. (2019). Mouse-Tracking. *A Handbook of Process Tracing Methods; Routledge: Abingdon, UK*, 111–130

[CR29] Kucina, T., Wells, L., Lewis, I., de Salas, K., Kohl, A., Palmer, M. A., ... & Heathcote, A. (2023). Calibration of cognitive tests to address the reliability paradox for decision-conflict tasks. *Nature Communications*, *14*(1), 2234. 10.1038/s41467-023-37777-2 PMC10115879 37076456

[CR30] Lane, A. E., & Ziviani, J. M. (2010). Factors influencing skilled use of the computer mouse by school-aged children. *Computers & Education,**55*(3), 1112–1122.

[CR31] Lerche, V., & Voss, A. (2017). Retest reliability of the parameters of the Ratcliff diffusion model. *Psychological Research Psychologische Forschung,**81*, 629–652. 27107855 10.1007/s00426-016-0770-5

[CR32] MacLeod, J. W., Lawrence, M. A., McConnell, M. M., Eskes, G. A., Klein, R. M., & Shore, D. I. (2010). Appraising the ANT: Psychometric and theoretical considerations of the Attention Network Test. *Neuropsychology,**24*(5), 637–651. 20804252 10.1037/a0019803

[CR33] Mayr, U., Awh, E., & Laurey, P. (2003). Conflict adaptation effects in the absence of executive control. *Nature Neuroscience,**6*(5), 450–452. 12704394 10.1038/nn1051

[CR34] Miller, J., & Ulrich, R. (2013). Mental chronometry and individual differences: Modeling reliabilities and correlations of reaction time means and effect sizes. *Psychonomic Bulletin & Review,**20*(5), 819–858. 23955122 10.3758/s13423-013-0404-5

[CR35] Moher, J., & Song, J. H. (2013). Context-dependent sequential effects of target selection for action. *Journal of Vision,**13*(8), 1–10. 10.1167/13.8.10 10.1167/13.8.10 PMC3711468 23847303

[CR36] Moretti, L., Koch, I., Hornjak, R., & von Bastian, C. C. (2023). Quality over quantity: Focusing on high-conflict trials to improve the reliability and validity of attentional control measures. Preprint. PsyArXiv, October 27, 2023. 10.31234/osf.io/ndjy6 10.1037/xlm0001466 39946591

[CR37] Nieuwenhuis, S., Stins, J. F., Posthuma, D., Polderman, T. J., Boomsma, D. I., & de Geus, E. J. (2006). Accounting for sequential trial effects in the flanker task: Conflict adaptation or associative priming? *Memory & Cognition,**34*(6), 1260–1272. 17225507 10.3758/bf03193270

[CR38] Nunnally, J. C. (1964). *Educational measurement and evaluation*. McGraw-Hill.

[CR39] Parsons, S. (2021). Splithalf: Robust estimates of split-half reliability. *Journal of Open Source Software,**6*(60), 3041.

[CR40] Pfister, R., & Foerster, A. (2022). How to measure post-error slowing: The case of pre-error speeding. *Behavior Research Methods, 54*(1), 435–443. 10.3758/s13428-021-01631-4 PMC8863758 34240334

[CR41] Price, R. B., Brown, V., & Siegle, G. J. (2019). Computational modeling applied to the dot-probe task yields improved reliability and mechanistic insights. *Biological Psychiatry, 85*(7), 606–612. 10.1016/j.biopsych.2018.09.022 PMC6420394 30449531

[CR42] Rappaport, B. I., Shankman, S. A., Glazer, J. E., Buchanan, S. N., Weinberg, A., & Letkiewicz, A. M. (2025). Psychometrics of driftdiffusion model parameters derived from the Eriksen flanker task: Reliability and validity in two independent samples. *Cognitive, Affective, & Behavioral Neuroscience, 25*, 311–328. 10.3758/s13415-024-01222-8 PMC11908889 39443415

[CR43] Ratcliff, R., & Rouder, J. N. (1998). Modeling response times for two-choice decisions. *Psychological Science,**9*(5), 347–356.

[CR44] Ratcliff, R., Smith, P. L., Brown, S. D., & McKoon, G. (2016). Diffusion decision model: Current issues and history. *Trends in Cognitive Sciences,**20*(4), 260–281. 26952739 10.1016/j.tics.2016.01.007 PMC4928591

[CR45] Rey-Mermet, A., Gade, M., & Oberauer, K. (2018). Should we stop thinking about inhibition? Searching for individual and age differences in inhibition ability. *Journal of Experimental Psychology: Learning, Memory, and Cognition,**44*(4), 501–526. 28956944 10.1037/xlm0000450

[CR46] Rouder, J. N., & Haaf, J. M. (2019). A psychometrics of individual differences in experimental tasks. *Psychonomic Bulletin & Review,**26*(2), 452–467. 30911907 10.3758/s13423-018-1558-y

[CR47] Rouder, J. N., & Mehrvarz, M. (2023). Hierarchical-model insights for planning and interpreting individual-difference studies of cognitive abilities. Pre-Print, OSF.

[CR48] Scherbaum, S., & Dshemuchadse, M. (2020). Psychometrics of the continuous mind: Measuring cognitive sub-processes via mouse tracking. *Memory & Cognition,**48*, 436–454. 31721062 10.3758/s13421-019-00981-x

[CR49] Schönbrodt, F. D., & Perugini, M. (2013). At what sample size do correlations stabilize? *Journal of Research in Personality,**47*(5), 609–612.

[CR50] Simon, J. R. (1969). Reactions toward the source of stimulation. *Journal of Experimental Psychology,**81*(1), 174–176. 10.1037/h0027448 5812172 10.1037/h0027448

[CR51] Smith, K. A., Morrison, S., Henderson, A. M., & Erb, C. D. (2022). Moving beyond response times with accessible measures of manual dynamics. *Scientific Reports,**12*(1), 19065. 36351962 10.1038/s41598-022-20579-9 PMC9646795

[CR52] Stoffels, E. J., & Van der Molen, M. W. (1988). Effects of visual and auditory noise on visual choice reaction time in a continuous-flow paradigm. *Perception & Psychophysics,**44*, 7–14. 3405732 10.3758/bf03207468

[CR53] Stroop, J. R. (1935). Studies of interference in serial verbal reactions. *Journal of Experimental Psychology, 18*(6), 643–662.

[CR54] Taylor, B. K., Frenzel, M. R., Eastman, J. A., Wiesman, A. I., Wang, Y. P., Calhoun, V. D., … Wilson, T. W. (2022). Reliability of the NIH toolbox cognitive battery in children and adolescents: A 3-year longitudinal examination. *Psychological Medicine,**52*(9), 1718–1727. 10.1017/S0033291720003487 33032665 10.1017/S0033291720003487 PMC8589010

[CR55] Unsworth, N., & Miller, A. L. (2024). The importance of conative factors for individual differences in attention control. *Journal of Experimental Psychology: Learning, Memory, and Cognition,**50*(9), 1361–1384. 10.1037/xlm0001356 38913727 10.1037/xlm0001356

[CR56] Waszak, F., Li, S. C., & Hommel, B. (2010). The development of attentional networks: Cross-sectional findings from a life span sample. *Developmental Psychology,**46*(2), 337. 20210494 10.1037/a0018541

[CR57] Wiecki, T. V., Sofer, I., & Frank, M. J. (2013). HDDM: Hierarchical Bayesian estimation of the drift-diffusion model in Python. *Frontiers in Neuroinformatics, 7*, 14. 10.3389/fninf.2013.00014 PMC3731670 23935581

[CR58] Zelazo, P. D., Anderson, J. E., Richler, J., Wallner-Allen, K., Beaumont, J. L., Conway, K. P., … Weintraub, S. (2014). NIH Toolbox Cognition Battery (CB): Validation of executive function measures in adults. *Journal of the International Neuropsychological Society,**20*(6), 620–629. 10.1017/S1355617714000472 24960301 10.1017/S1355617714000472 PMC4601803

