# Supplementary material for: Moving beyond button presses to enhance the reliability of congruency tasks
Source: Behav Res Methods. 2025 Jul 2;57(8):215. doi: 10.3758/s13428-025-02740-0 (PMC12222348; doi:10.3758/s13428-025-02740-0)
Supplement: Supplementary file 1 — Supplementary file1 (DOCX 244 KB) [file 13428_2025_2740_MOESM1_ESM.docx]

**Supplementary Materials for *Moving Beyond Button Presses to Enhance the Reliability of Congruency Tasks***

**Section 1.**

Descriptive statistics for the datasets featured in Study 1, displaying average performance across all trials (congruent and incongruent).

**Table S1**

*Descriptive Statistics for Response Time, Initiation Time, Movement Time, Curvature, and Error Rates for Flanker Task Datasets*

| Experiment | Age Group | Measure | M | SD | Min | Max | Skew | Kurtosis |
| --- | --- | --- | --- | --- | --- | --- | --- | --- |
| Erb et al. (2016, Exp. 2) | Young adults | RT | 847 | 122 | 619 | 1305 | 1.27 | 3.33 |
|  |  | IT | 431 | 88 | 305 | 775 | 1.54 | 4.15 |
|  |  | MT | 416 | 55 | 282 | 529 | -0.19 | 0.21 |
|  |  | CURV | 0.125 | 0.040 | 0.061 | 0.214 | 0.48 | -0.64 |
|  |  | Error Rate (%) | 0.5 | 0.6 | 0.0 | 2.8 | 2.06 | 5.83 |
| Erb et al. (2018, Exp. 1) | 5- to 10-year-olds | RT | 1212 | 380 | 827 | 3129 | 2.70 | 9.76 |
|  |  | IT | 666 | 224 | 344 | 1582 | 1.6 | 3.55 |
|  |  | MT | 546 | 176 | 344 | 1547 | 3.60 | 16.58 |
|  |  | CURV | 0.105 | 0.04 | 0.032 | 0.249 | 0.84 | 1.49 |
|  |  | Error Rate (%) | 1.4 | 2.0 | 0 | 8.2 | 2.13 | 3.76 |
| Erb et al. (2018, Exp. 2) | Young adults | RT | 815 | 89 | 671 | 981 | 0.23 | -1.07 |
|  |  | IT | 396 | 57 | 316 | 526 | 0.60 | -0.46 |
|  |  | MT | 419 | 55 | 314 | 530 | 0.15 | -0.62 |
|  |  | CURV | 0.070 | 0.032 | 0.003 | 0.119 | -0.22 | -0.65 |
|  |  | Error Rate (%) | 0.1 | 0.2 | 0 | 1.1 | 3.03 | 9.02 |
| Erb & Marcovitch (2018) | 6- to 8-year-olds | RT | 1300 | 279 | 840 | 2054 | 0.78 | 0.03 |
|  |  | IT | 724 | 212 | 394 | 1380 | 0.86 | 0.60 |
|  |  | MT | 576 | 104 | 447 | 837 | 1.21 | 0.70 |
|  |  | CURV | 0.114 | 0.038 | 0.049 | 0.209 | 0.78 | 0.10 |
|  |  | Error Rate (%) | 1.9 | 2.2 | 0 | 8.9 | 1.76 | 2.95 |
|  | 10- to 12-year-olds | RT | 903 | 136 | 690 | 1551 | 2.43 | 9.68 |
|  |  | IT | 445 | 107 | 290 | 1004 | 3.20 | 14.62 |
|  |  | MT | 459 | 50 | 339 | 547 | -0.24 | -0.51 |
|  |  | CURV | 0.091 | 0.028 | 0.026 | 0.156 | -0.14 | 0.04 |
|  |  | Error Rate (%) | 0.4 | 0.7 | 0 | 2.9 | 1.83 | 2.93 |
|  | Young adults | RT | 815 | 89 | 691 | 1044 | 1.00 | 0.58 |
|  |  | IT | 375 | 66 | 286 | 537 | 0.85 | 0 |
|  |  | MT | 440 | 53 | 340 | 542 | 0.21 | -0.76 |
|  |  | CURV | 0.054 | 0.027 | -0.002 | 0.11 | 0.16 | -0.54 |
|  |  | Error Rate (%) | 0.2 | 0.5 | 0.0 | 2.4 | 3.00 | 10.18 |
| Erb et al. (2020) | Older adults | RT | 937 | 115 | 703 | 1219 | 0.40 | -0.19 |
|  |  | IT | 470 | 96 | 294 | 735 | 0.46 | 0.09 |
|  |  | MT | 468 | 36 | 404 | 547 | 0.13 | -0.75 |
|  |  | CURV | 0.077 | 0.032 | 0.026 | 0.163 | 0.98 | 0.91 |
|  |  | Error Rate (%) | 0.2 | 0.4 | 0 | 1.4 | 2.42 | 4.39 |
| Erb et al. (2021) | Young adults | RT | 810 | 88 | 643 | 1068 | 0.60 | 0.35 |
|  |  | IT | 363 | 59 | 241 | 530 | 0.56 | -0.14 |
|  |  | MT | 447 | 52 | 335 | 613 | 0.26 | -0.10 |
|  |  | CURV | 0.063 | 0.027 | 0.006 | 0.142 | 0.21 | -0.28 |
|  |  | Error Rate (%) | 0.1 | 0.5 | 0 | 5.3 | 9.78 | 105.72 |
| Smith et al. (2022) | 6- to 8-year-olds | RT | 1175 | 231 | 879 | 1905 | 1.36 | 1.67 |
|  |  | IT | 730 | 153 | 414 | 975 | -0.22 | -0.75 |
|  |  | MT | 445 | 160 | 243 | 1014 | 1.95 | 4.20 |
|  |  | Error Rate (%) | 5.3 | 7.0 | 0 | 32.2 | 2.57 | 6.57 |
|  | Young adults | RT | 650 | 74 | 510 | 860 | 0.42 | 0.16 |
|  |  | IT | 360 | 47 | 263 | 470 | 0.09 | -0.18 |
|  |  | MT | 290 | 63 | 171 | 441 | 0.26 | -0.53 |
|  |  | Error Rate (%) | 0.6 | 0.7 | 0 | 3.0 | 1.48 | 1.86 |

**Table S2**

*Descriptive Statistics for Response Time, Initiation Time, Movement Time, Curvature, and Error Rates for Stroop and Simon Task Datasets*

| Experiment | Task | Age Group | Measure | M | SD | Min | Max | Skew | Kurtosis |
| --- | --- | --- | --- | --- | --- | --- | --- | --- | --- |
| Erb et al. (2016, Exp. 1) | Stroop | Young adults | RT | 879 | 105 | 716 | 1153 | 0.77 | 0.33 |
|  |  |  | IT | 459 | 71 | 343 | 609 | 0.34 | -0.49 |
|  |  |  | MT | 420 | 63 | 325 | 570 | 0.90 | 0.15 |
|  |  |  | CURV | 0.159 | 0.047 | 0.093 | 0.277 | 0.97 | 0.22 |
|  |  |  | Error Rate (%) | 3.5 | 2.7 | 0 | 9.9 | 0.82 | -0.19 |
| Erb & Marcovitch (2019) | Simon | 6- to 8-year-olds | RT | 1267 | 194 | 909 | 1693 | 0.10 | -0.39 |
|  |  |  | IT | 684 | 140 | 413 | 983 | 0.19 | -0.45 |
|  |  |  | MT | 583 | 90 | 439 | 890 | 0.96 | 2.03 |
|  |  |  | CURV | 0.168 | 0.051 | 0.087 | 0.319 | 0.73 | 0.58 |
|  |  |  | Error Rate (%) | 3.6 | 4.1 | 0 | 19.3 | 2.03 | 4.40 |
|  |  | 10- to 12-year-olds | RT | 963 | 91 | 739 | 1136 | -0.18 | -0.27 |
|  |  |  | IT | 496 | 70 | 360 | 613 | 0.03 | -0.92 |
|  |  |  | MT | 467 | 70 | 336 | 633 | 0.03 | -0.56 |
|  |  |  | CURV | 0.104 | 0.047 | 0.008 | 0.208 | 0.22 | -0.04 |
|  |  |  | Error Rate (%) | 0.8 | 1.1 | 0 | 4.2 | 1.67 | 2.07 |
|  |  | Young adults | RT | 819 | 96 | 649 | 1053 | 0.48 | -0.16 |
|  |  |  | IT | 387 | 75 | 288 | 547 | 0.69 | -0.48 |
|  |  |  | MT | 431 | 44 | 351 | 528 | 0.54 | -0.11 |
|  |  |  | CURV | 0.068 | 0.028 | 0 | 0.141 | 0.11 | 0.45 |
|  |  |  | Error Rate (%) | 0.2 | 0.4 | 0 | 1.5 | 2.01 | 3.59 |

**Section 2.**

The datasets were filtered by excluding all trials featuring RTs under 200 ms or over 2.5 for adult participants and all trials featuring RTs under 200 ms or over 4 seconds for child participants. The mean and standard deviation of each participants' overall response time on each condition were then calculated. Any trials featuring a RT greater than 3 SDs from an individual's own mean RT for each condition were considered outliers and subsequently removed. Log-transformations were then applied by multiplying each RT, IT, and MT value by the natural logarithm (ln). Given (a) that it is inappropriate to perform a log transformation on a negative number, and (b) that reach curvatures can be positive or negative, a constant of 1 was added to each CURV value to ensure that all values were positive before the log transformation was applied.

**Table S3**

*Descriptive Statistics for Congruency Effects observed in Filtered Response Time, Initiation Time, Movement Time, and Curvature for Flanker Task Datasets*

| Experiment | Age Group | Measure | M | SD | Min | Max | Skew | Kurtosis |
| --- | --- | --- | --- | --- | --- | --- | --- | --- |
| Erb et al. (2016, Exp. 2) | Young adults | RT | 30 | 16 | 4 | 94 | 1.58 | 4.21 |
|  |  | IT | 16 | 15 | -13 | 68 | 0.99 | 2.24 |
|  |  | MT | 14 | 11 | -5 | 37 | 0.28 | -0.66 |
|  |  | CURV | 0.038 | 0.032 | -0.028 | 0.109 | 0.14 | -0.35 |
| Erb et al. (2018, Exp. 1) | 5- to 10-year-olds | RT | 90 | 140 | -150 | 768 | 3.29 | 12.79 |
|  |  | IT | 39 | 94 | -246 | 434 | 1.88 | 8.24 |
|  |  | MT | 51 | 63 | -69 | 334 | 2.32 | 7.60 |
|  |  | CURV | 0.052 | 0.038 | -0.004 | 0.139 | 0.59 | -0.66 |
| Erb et al. (2018, Exp. 2) | Young adults | RT | 42 | 16 | 12 | 80 | 0.19 | 0.11 |
|  |  | IT | 33 | 20 | 6 | 83 | 0.63 | -0.19 |
|  |  | MT | 9 | 11 | -5 | 28 | 0.47 | -1.03 |
|  |  | CURV | 0.038 | 0.033 | -0.004 | 0.100 | 0.57 | -1.00 |
| Erb & Marcovitch (2018) | 6- to 8-year-olds | RT | 321 | 270 | 41 | 1161 | 1.39 | 1.26 |
|  |  | IT | 179 | 184 | -50 | 791 | 1.71 | 2.28 |
|  |  | MT | 142 | 132 | 8 | 607 | 1.69 | 3.00 |
|  |  | CURV | 0.089 | 0.062 | -0.004 | 0.295 | 1.35 | 1.80 |
|  | 10- to 12-year-olds | RT | 70 | 44 | -3 | 234 | 1.74 | 3.80 |
|  |  | IT | 38 | 30 | -11 | 120 | 0.77 | 0.24 |
|  |  | MT | 32 | 27 | -5 | 125 | 1.16 | 1.69 |
|  |  | CURV | 0.065 | 0.035 | 0.004 | 0.171 | 0.42 | 0.27 |
|  | Young adults | RT | 47 | 28 | 7 | 168 | 2.2 | 6.64 |
|  |  | IT | 37 | 28 | 3 | 156 | 2.19 | 6.27 |
|  |  | MT | 10 | 13 | -8 | 59 | 1.58 | 2.85 |
|  |  | CURV | 0.041 | 0.031 | 0.002 | 0.138 | 1.20 | 1.41 |
| Erb et al. (2020) | Older adults | RT | 82 | 44 | 22 | 290 | 2.56 | 9.50 |
|  |  | IT | 61 | 42 | 3 | 210 | 1.19 | 1.95 |
|  |  | MT | 22 | 20 | -9 | 81 | 1.04 | 0.70 |
|  |  | CURV | 0.05 | 0.04 | 0.002 | 0.181 | 1.30 | 1.32 |
| Erb et al. (2021) | Young adults | RT | 40 | 21 | 5 | 189 | 3.24 | 18.88 |
|  |  | IT | 27 | 22 | -14 | 156 | 1.86 | 8.24 |
|  |  | MT | 13 | 13 | -12 | 65 | 0.8 | 1.1 |
|  |  | CURV | 0.049 | 0.033 | -0.004 | 0.145 | 0.49 | -0.56 |
| Smith et al. (2022) | 6- to 8-year-olds | RT | 292 | 261 | 66 | 1322 | 2.17 | 4.87 |
|  |  | IT | 149 | 130 | -48 | 554 | 1.16 | 1.17 |
|  |  | MT | 143 | 204 | -78 | 1083 | 2.91 | 9.51 |
|  | Young adults | RT | 55 | 17 | 23 | 104 | 0.34 | -0.03 |
|  |  | IT | 27 | 16 | -4 | 63 | 0.29 | -0.43 |
|  |  | MT | 29 | 18 | 2 | 82 | 0.88 | 0.68 |

**Table S4**

*Descriptive Statistics for Congruency Effects observed in Filtered Response Time, Initiation Time, Movement Time and Curvature for Stroop and Simon Task Datasets*

| Experiment | Task | Age Group | Measure | M | SD | Min | Max | Skew | Kurtosis |
| --- | --- | --- | --- | --- | --- | --- | --- | --- | --- |
| Erb et al. (2016, Exp. 1) | Stroop | Young adults | RT | 53 | 34 | 16 | 128 | 0.87 | -0.46 |
|  |  |  | IT | 27 | 26 | -3 | 103 | 1.46 | 1.60 |
|  |  |  | MT | 27 | 20 | 2 | 77 | 0.76 | -0.29 |
|  |  |  | CURV | 0.037 | 0.039 | -0.013 | 0.123 | 0.76 | -0.47 |
| Erb & Marcovitch (2019) | Simon | 6- to 8-year-olds | RT | 52 | 65 | -100 | 219 | 0.34 | 0.24 |
|  |  |  | IT | 11 | 37 | -50 | 170 | 2.27 | 7.84 |
|  |  |  | MT | 41 | 55 | -76 | 171 | 0.45 | -0.04 |
|  |  |  | CURV | 0.097 | 0.079 | -0.023 | 0.273 | 0.63 | -0.39 |
|  |  | 10- to 12-year-olds | RT | 25 | 26 | -26 | 94 | 0.21 | 0.29 |
|  |  |  | IT | 11 | 20 | -36 | 52 | -0.11 | -0.36 |
|  |  |  | MT | 14 | 18 | -33 | 54 | 0.09 | 0.51 |
|  |  |  | CURV | 0.04 | 0.033 | -0.023 | 0.113 | 0.35 | -0.47 |
|  |  | Young adults | RT | 15 | 19 | -11 | 90 | 1.8 | 5.49 |
|  |  |  | IT | 8 | 16 | -13 | 79 | 2.45 | 9.07 |
|  |  |  | MT | 7 | 8 | -9 | 23 | -0.03 | -0.61 |
|  |  |  | CURV | 0.022 | 0.025 | -0.017 | 0.095 | 0.90 | 0.47 |

**Table S5**

*Descriptive Statistics for Congruency Effects observed in Filtered, Log-Transformed Response Time, Initiation Time, Movement Time, and Curvature for Flanker Task Datasets*

| Experiment | Age Group | Measure | M | SD | Min | Max | Skew | Kurtosis |
| --- | --- | --- | --- | --- | --- | --- | --- | --- |
| Erb et al. (2016, Exp. 2) | Young adults | RT | 0.035 | 0.015 | 0.005 | 0.075 | 0.41 | 0.06 |
|  |  | IT | 0.032 | 0.032 | -0.057 | 0.101 | -0.56 | 0.88 |
|  |  | MT | 0.034 | 0.026 | -0.012 | 0.112 | 0.72 | 0.64 |
|  |  | CURV | 0.027 | 0.025 | -0.026 | 0.086 | 0.14 | -0.18 |
| Erb et al. (2018, Exp. 1) | 5- to 10-year-olds | RT | 0.067 | 0.066 | -0.069 | 0.383 | 2.19 | 7.99 |
|  |  | IT | 0.047 | 0.081 | -0.197 | 0.320 | 0.31 | 1.83 |
|  |  | MT | 0.073 | 0.066 | -0.057 | 0.337 | 1.2 | 3.12 |
|  |  | CURV | 0.041 | 0.030 | -0.003 | 0.144 | 0.63 | -0.52 |
| Erb et al. (2018, Exp. 2) | Young adults | RT | 0.052 | 0.018 | 0.013 | 0.092 | -0.09 | -0.05 |
|  |  | IT | 0.081 | 0.041 | 0.021 | 0.174 | 0.41 | -0.58 |
|  |  | MT | 0.02 | 0.026 | -0.012 | 0.078 | 0.64 | -0.69 |
|  |  | CURV | 0.033 | 0.029 | -0.004 | 0.087 | 0.51 | -1.07 |
| Erb & Marcovitch (2018) | 6- to 8-year-olds | RT | 0.227 | 0.153 | 0.033 | 0.623 | 1.09 | 0.39 |
|  |  | IT | 0.205 | 0.166 | -0.147 | 0.659 | 1.12 | 1.27 |
|  |  | MT | 0.184 | 0.143 | 0.008 | 0.697 | 1.35 | 2.41 |
|  |  | CURV | 0.071 | 0.048 | -0.005 | 0.227 | 1.24 | 1.51 |
|  | 10- to 12-year-olds | RT | 0.077 | 0.041 | -0.001 | 0.2 | 1.19 | 1.62 |
|  |  | IT | 0.078 | 0.058 | -0.052 | 0.239 | 0.17 | 0.23 |
|  |  | MT | 0.061 | 0.050 | -0.011 | 0.187 | 0.75 | -0.08 |
|  |  | CURV | 0.055 | 0.029 | 0.004 | 0.137 | 0.34 | -0.05 |
|  | Young adults | RT | 0.057 | 0.027 | 0.009 | 0.159 | 1.34 | 2.89 |
|  |  | IT | 0.09 | 0.051 | 0.01 | 0.267 | 1.10 | 1.91 |
|  |  | MT | 0.021 | 0.028 | -0.015 | 0.117 | 1.40 | 1.83 |
|  |  | CURV | 0.037 | 0.027 | 0.002 | 0.118 | 1.08 | 1.06 |
| Erb et al. (2020) | Older adults | RT | 0.086 | 0.037 | 0.026 | 0.245 | 1.92 | 6.40 |
|  |  | IT | 0.117 | 0.065 | 0.007 | 0.279 | 0.44 | -0.17 |
|  |  | MT | 0.041 | 0.04 | -0.017 | 0.149 | 0.95 | 0.17 |
|  |  | CURV | 0.044 | 0.033 | 0.002 | 0.151 | 1.2 | 1.07 |
| Erb et al. (2021) | Young adults | RT | 0.049 | 0.022 | 0.007 | 0.192 | 2.58 | 13.14 |
|  |  | IT | 0.067 | 0.047 | -0.052 | 0.288 | 0.74 | 2.74 |
|  |  | MT | 0.027 | 0.027 | -0.033 | 0.134 | 0.75 | 1.12 |
|  |  | CURV | 0.044 | 0.029 | -0.004 | 0.124 | 0.42 | -0.69 |
| Smith et al. (2022) | 6- to 8-year-olds | RT | 0.224 | 0.141 | 0.066 | 0.71 | 1.53 | 2.16 |
|  |  | IT | 0.176 | 0.126 | -0.04 | 0.509 | 0.59 | -0.03 |
|  |  | MT | 0.223 | 0.221 | -0.142 | 1.16 | 1.89 | 5.58 |
|  | Young adults | RT | 0.087 | 0.028 | 0.034 | 0.19 | 0.89 | 2.51 |
|  |  | IT | 0.068 | 0.038 | -0.023 | 0.143 | -0.22 | -0.22 |
|  |  | MT | 0.097 | 0.058 | 0.002 | 0.299 | 0.91 | 1.41 |

**Table S6**

*Descriptive Statistics for Congruency Effects observed in Filtered, Log-Transformed Response Time, Initiation Time, Movement Time and Curvature for Stroop and Simon Task Datasets*

| Experiment | Task | Age Group | Measure | M | SD | Min | Max | Skew | Kurtosis |
| --- | --- | --- | --- | --- | --- | --- | --- | --- | --- |
| Erb et al. (2016, Exp. 1) | Stroop | Young adults | RT | 0.058 | 0.034 | 0.021 | 0.139 | 0.93 | -0.07 |
|  |  |  | IT | 0.050 | 0.047 | -0.029 | 0.164 | 0.90 | 0.47 |
|  |  |  | MT | 0.056 | 0.039 | 0.006 | 0.139 | 0.61 | -0.75 |
|  |  |  | CURV | 0.023 | 0.028 | -0.016 | 0.088 | 0.78 | -0.28 |
| Erb & Marcovitch (2019) | Simon | 6- to 8-year-olds | RT | 0.045 | 0.05 | -0.062 | 0.166 | 0.2 | -0.23 |
|  |  |  | IT | 0.017 | 0.044 | -0.043 | 0.183 | 1.60 | 4.15 |
|  |  |  | MT | 0.064 | 0.085 | -0.115 | 0.278 | 0.39 | 0.19 |
|  |  |  | CURV | 0.077 | 0.061 | -0.018 | 0.217 | 0.60 | -0.38 |
|  |  | 10- to 12-year-olds | RT | 0.027 | 0.027 | -0.024 | 0.089 | -0.03 | -0.27 |
|  |  |  | IT | 0.023 | 0.041 | -0.073 | 0.110 | -0.39 | -0.05 |
|  |  |  | MT | 0.025 | 0.034 | -0.084 | 0.089 | -0.57 | 1.39 |
|  |  |  | CURV | 0.033 | 0.027 | -0.02 | 0.096 | 0.34 | -0.28 |
|  |  | Young adults | RT | 0.019 | 0.021 | -0.014 | 0.097 | 1.34 | 3.69 |
|  |  |  | IT | 0.02 | 0.035 | -0.04 | 0.149 | 1.20 | 3.27 |
|  |  |  | MT | 0.016 | 0.017 | -0.022 | 0.049 | -0.15 | -0.64 |
|  |  |  | CURV | 0.019 | 0.022 | -0.016 | 0.078 | 0.80 | 0.17 |

Reliability analyses for the filtered datasets (Table S7) and the log-transformed and filtered datasets (Table S8).

**Table S7**
*List of Experiments with Split-half Spearman-Brown Corrected Reliability Estimates for Filtered Response Time (RT), Initiation Time (IT), Movement Time (MT), and Movement Curvature (CURV) Congruency Effects*

| Experiment | Task | Age Group | *N* | RT | IT | MT | CURV |
| --- | --- | --- | --- | --- | --- | --- | --- |
| Erb et al. (2016, Exp. 2) | Flanker | Young adults | 40 | .61  [.23 - .81] | .69  [.40 - .85] | .58  [.30 - .77] | .65  [.44 - .81] |
| Erb et al. (2018, Exp. 1) | Flanker | 5- to 10-year-olds | 60 | .79  [.58 – .91] | .64  [.17 – .85] | .44  [-.40 – .81] | .28  [-.20 – .57] |
| Erb et al. (2018, Exp. 2) | Flanker | Young adults | 24 | .69  [.42 – .86] | .87  [.74 – .94] | .72  [.50 – .87] | .93  [.88 – .97] |
| Erb & Marcovitch (2018) | Flanker | 6- to 8-year-olds | 45 | .96  [.94 – .98] | .94  [.89 – .97] | .91  [.84 – .95] | .83  [.73 – .90] |
|  |  | 10- to 12-year-olds | 45 | .79  [.61 – .89] | .63  [.33 – .81] | .74  [.60 – .85] | .74  [.58 – .85] |
|  |  | Young adults | 45 | .82  [.70 – .90] | .88  [.78 – .94] | .71  [.56 – .82] | .88  [.81 – .93] |
| Erb et al. (2020) | Flanker | Older adults | 45 | .90  [.81 – .95] | .91  [.82 – .96] | .77  [.63 – .87] | .93  [.89 – .96] |
| Erb et al. (2021) | Flanker | Young adults | 135 | .82  [.77 – .87] | .89  [.85 – .92] | .77  [.71 – .83] | .93  [.90 – .94] |
| Smith et al. (2022) | Flanker | 6- to 8-year-olds | 45 | .96  [.91 – .98] | .91  [.84 – .95] | .96  [.91 – .98] | N/A |
|  |  | Young adults | 45 | .62  [.41 – .78] | .80  [.69 – .88] | .70  [.53 – .83] | N/A |
| Erb et al. (2016, Exp. 1) | Stroop | Young adults | 24 | .89  [.79 – .95] | .89  [.81 – .95] | .85  [.69 – .94] | .76  [.56 – .89] |
| Erb & Marcovitch (2019) | Simon | 6- to 8-year-olds | 36 | .59  [.31 – .79] | .04  [-.44 – .50] | .66  [.44 – .83] | .85  [.75 – .91] |
|  |  | 10- to 12-year-olds | 36 | .43  [.10 – .68] | .23  [-.20 – .57] | .41  [.06 – .67] | .55  [.27 – .75] |
|  |  | Young adults | 36 | .70  [.51 – .83] | .72  [.55 – .85] | .25  [-.15 – .59] | .76  [.59 – .88] |

Yellow, light green and dark green cells indicate Spearman-Brown corrected split-half reliability estimates of .70 to .79 (acceptable), .80 to .89 (good), and .90 to 1.0 (excellent). respectively. 95% confidence intervals are presented in brackets.

**Table S8**
*List of Experiments with Split-half Spearman-Brown Corrected Reliability Estimates for Filtered and Log-Transformed Response Time (RT), Initiation Time (IT), Movement Time (MT), and Movement Curvature (CURV) Congruency Effects*

| Experiment | Task | Age Group | *N* | RT | IT | MT | CURV |
| --- | --- | --- | --- | --- | --- | --- | --- |
| Erb et al. (2016, Exp. 2) | Flanker | Young adults | 40 | .49  [.16 - .71] | .60  [.36 - .77] | .64  [.43 - .79] | .63  [.42 - .79] |
| Erb et al. (2018, Exp. 1) | Flanker | 5- to 10-year-olds | 60 | .62  [.39 – .78] | .39  [.03 – .63] | .31  [-.27 – .64] | .62  [.39 – .78] |
| Erb et al. (2018, Exp. 2) | Flanker | Young adults | 24 | .68  [.43 – .85] | .82  [.67 – .91] | .75  [.56 – .88] | .93  [.87 – .97] |
| Erb & Marcovitch (2018) | Flanker | 6- to 8-year-olds | 45 | .95  [.93 – .97] | .89  [.82 – .93] | .88  [.81 – .93] | .81  [.70 – .88] |
|  |  | 10- to 12-year-olds | 45 | .76  [.64 – .86] | .51  [.25 – .72] | .71  [.57 – .83] | .73  [.58 – .84] |
|  |  | Young adults | 45 | .74  [.60 – .85] | .76  [.63 – .87] | .68  [.52 – .80] | .87  [.80 – .93] |
| Erb et al. (2020) | Flanker | Older adults | 45 | .85  [.76 – .92] | .86  [.77 – .92] | .78  [.67 – .87] | .93  [.88 – .96] |
| Erb et al. (2021) | Flanker | Young adults | 135 | .78  [.72 – .83] | .78  [.71 – .82] | .77  [.71 – .82] | .92  [.90 – .94] |
| Smith et al. (2022) | Flanker | 6- to 8-year-olds | 45 | .92  [.87 – .96] | .83  [.72 – .91] | .91  [.85 – .95] | N/A |
|  |  | Young adults | 45 | .67  [.50 – .80] | .70  [.54 – .82] | .72  [.58 – .84] | N/A |
| Erb et al. (2016, Exp. 1) | Stroop | Young adults | 24 | .87  [.77 – .94] | .84  [.70 – .93] | .84  [.70 – .93] | .74  [.53 – .88] |
| Erb & Marcovitch (2019) | Simon | 6- to 8-year-olds | 36 | .63  [.40 – .80] | -.26  [-.58 – .21] | .71  [.53 – .84] | .84  [.74 – .91] |
|  |  | 10- to 12-year-olds | 36 | .44  [.13 – .68] | .25  [-.16 – .58] | .39  [.04 – .66] | .55  [.28 – .75] |
|  |  | Young adults | 36 | .67  [.48 – .81] | .51  [.15 – .75] | .29  [-.12 – .61] | .76  [.59 – .87] |

Yellow, light green and dark green cells indicate Spearman-Brown corrected split-half reliability estimates of .70 to .79 (acceptable), .80 to .89 (good), and .90 to 1.0 (excellent). respectively. 95% confidence intervals are presented in brackets.

**Section 3.**

**Table S9**

*Descriptive Statistics for Response Time, Initiation Time, and Movement Time for the Keyboard and Touchscreen Flanker Task Conditions*

| Task | Measure | M | SD | Min | Max | Skew | Kurtosis |
| --- | --- | --- | --- | --- | --- | --- | --- |
| Keyboard | RT | 596 | 82 | 478 | 878 | 1.1 | 1.83 |
|  | IT | 394 | 81 | 283 | 700 | 1.25 | 2.33 |
|  | MT | 203 | 56 | 112 | 397 | 1.23 | 2.51 |
|  | Error Rate (%) | 1.2 | 1.5 | 0 | 7.0 | 2.22 | 4.83 |
| Touchscreen | RT | 802 | 100 | 651 | 1199 | 1.5 | 3.69 |
|  | IT | 424 | 80 | 302 | 802 | 2.23 | 7.97 |
|  | MT | 378 | 68 | 240 | 643 | 1.06 | 3.03 |
|  | Error Rate (%) | 0.1 | 0.7 | 0 | 4.9 | 6.52 | 42.11 |

Next, we evaluated the correlations between each of the computed congruency effects (see Table S10), while acknowledging that these analyses should be interpreted with caution given our small sample size. The congruency effects observed in each measure were highly correlated across the tasks, with RT congruency effects correlating at .75, IT congruency effects correlating at .80, and MT congruency effects correlating at .58. Both conditions revealed a negative correlation between the IT and MT congruency effects, with the keyboard condition revealing a correlation of -.50 and the touchscreen condition revealing a correlation of -.50. These results demonstrate that individuals who tended to generate smaller congruency effects in IT also tended to generate larger congruency effects in MT.

**Table S10**

*Correlation Matrix for Response Time, Initiation Time, and Movement Time Congruency Effects for the Keyboard and Touchscreen Flanker Task Conditions*

|  | 1. | 2. | 3. | 4. | 5. | 6. |
| --- | --- | --- | --- | --- | --- | --- |
| 1. Keyboard RT | - |  |  |  |  |  |
| 2. Touchscreen RT | .75***  [.60 – .85] | - |  |  |  |  |
| 3. Keyboard IT | .69***  [.51 – .81] | .57***  [.35 – .73] | - |  |  |  |
| 4. Touchscreen IT | .64***  [.45 – .78] | .73***  [.56 – .83] | .80***  [.67 – .88] | - |  |  |
| 5. Keyboard MT | .28*  [.00 – .52] | .14  [-.14 – .40] | -.50***  [-.68 – -.26] | -.29*  [-.52 – -.01] | - |  |
| 6. Touchscreen MT | .03  [-.25 – .30] | .23  [-.05 – .48] | -.41***  [-.62 – -.15] | -.50***  [-.68 – -.26] | .58***  [.36 – .74] | - |

Note. ****p* < .001, ***p* < .01, **p* < .05. 95% confidence intervals are presented in brackets.

**Section 4.**

Results from 2 (Trial *n* congruency: C vs. I) × 2 (Trial *n*-1 congruency: c vs. i) × 2 (Response repetition type: alternation vs. repetition) ANOVAs featured in Study 2 of manuscript.

**KEYBOARD CONDITION**

**RT**

**
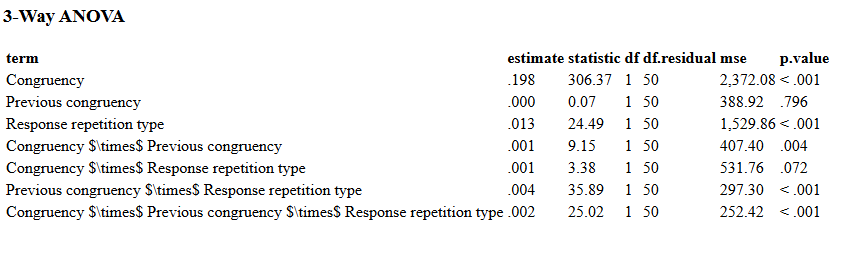
**

**IT**


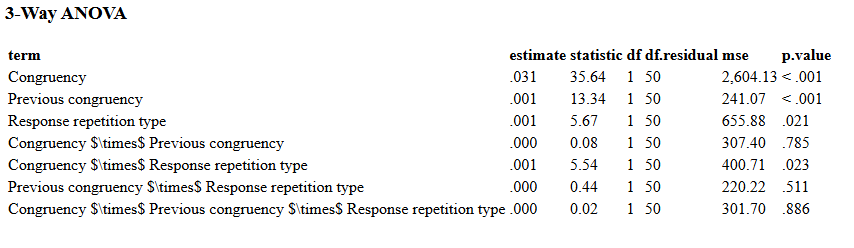


**MT**


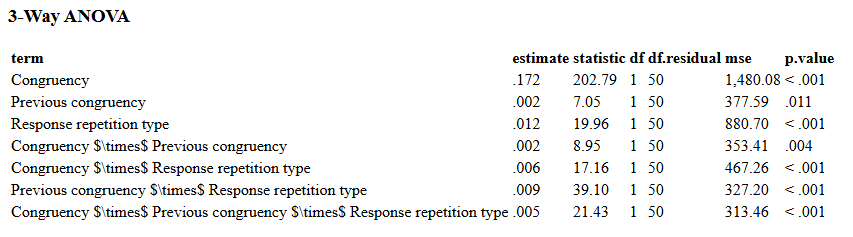


**TOUCHSCREEN CONDITION**

**RT**

**
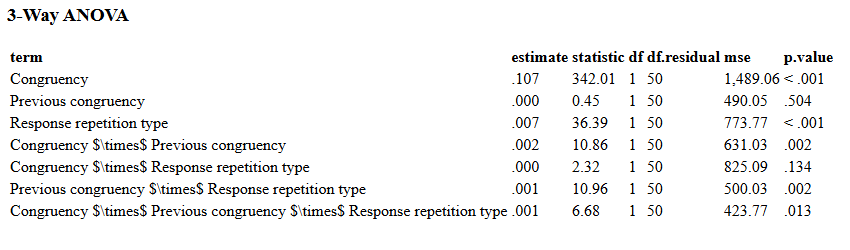
**

**IT**


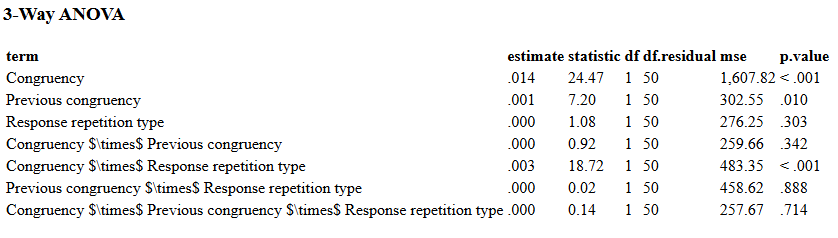


**MT**

**
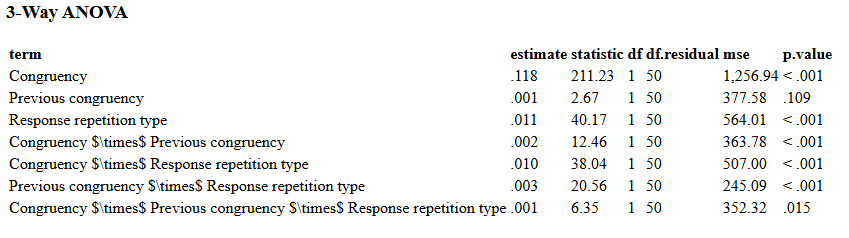
**
